# The relationship between hospital ownership, in-hospital mortality, and medical expenses: an analysis of three common conditions in China

**DOI:** 10.1186/s13690-023-01029-y

**Published:** 2023-02-10

**Authors:** Qingping Xue, Dong Roman Xu, Terence C. Cheng, Jay Pan, Winnie Yip

**Affiliations:** 1 School of Public Health, Chengdu Medical College, Chengdu, China; 2 Institute for Healthy Cities and West China Research Center for Rural Health Development, Sichuan University, Chengdu, China; 3 Center for World Health Organization Studies and Department of Health Management, School of Health Management of Southern Medical University, Guangzhou, China; 4 Acacia Lab for Implementation Research, SMU Institute for Global Health (SIGHT), Dermatology Hospital of Southern Medical University (SMU), Guangzhou, China; 5 Harvard TH Chan School of Public Health, Boston, USA; 6 HEOA Group, West China School of Public Health and West China Fourth Hospital, Sichuan University, Chengdu, China; 7 School of Public Administration, Sichuan University, Chengdu, China

**Keywords:** Private hospital, For-profit hospital, Quality of care, Medical expense, China

## Abstract

**Background:**

Private hospitals expanded rapidly in China since 2009 following its national health reform encouraging private investment in the hospital sector. Despite long-standing debates over the performance of different types of hospitals, empirical evidence under the context of developing countries remains scant. We investigated the disparities in health care quality and medical expenses among public, private not-for-profit, and private for-profit hospitals.

**Methods:**

A total of 64,171 inpatients (51,933 for pneumonia (PNA), 9,022 for heart failure (HF) and 3,216 for acute myocardial infarction (AMI)) who were admitted to 528 secondary hospitals in Sichuan province, China, during the fourth quarters of 2016, 2017, and 2018 were selected for this study. Multilevel logistic regressions and multilevel linear regressions were utilized to assess the relationship between hospital ownership types and in-hospital mortality, as well as medical expenses for PNA, HF, and AMI, after adjusting for relevant hospital and patient characteristics, respectively.

**Results:**

The private not-for-profit (adjusted OR, 1.69; 95% CI, 1.08, 2.64) and for-profit (adjusted OR, 1.67; 95% CI, 1.06, 2.62) hospitals showed higher in-hospital mortality than the public ones for PNA, but not for AMI and HF. No significant differences were found in medical expenses across hospital ownership types for AMI, but the private not-for-profit was associated with 9% higher medical expenses for treating HF, while private not-for-profit and for-profit hospitals were associated with 10% and 11% higher medical expenses for treating PNA than the public hospitals. No differences were found between the private not-for-profit and private for-profit hospitals both in in-hospital mortality and medical expenses across the three conditions.

**Conclusion:**

The public hospitals had at least equal or even higher healthcare quality and lower medical expenses than the private ones in China, while private not-for-profit and for-profit hospitals had similar performances in these aspects. Our results added evidences on hospitals’ performances among different ownership types under China’s context, which has great potential to inform the optimization of healthcare systems implemented among developing countries confronted with similar challenges.

**Supplementary Information:**

The online version contains supplementary material available at 10.1186/s13690-023-01029-y.

## Background

**Fig. 1:**
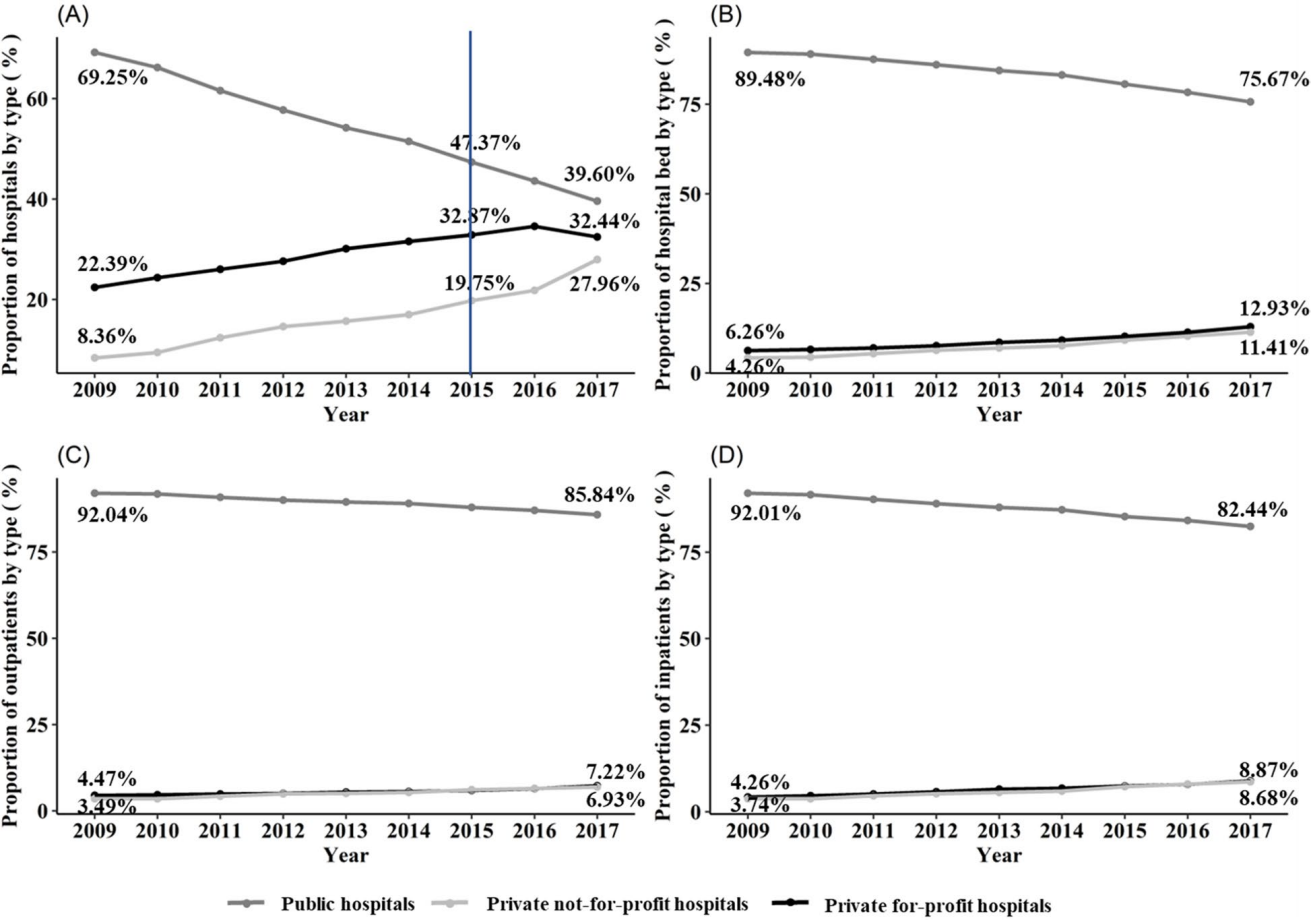
Trends in proportions of hospital number, beds, inpatients and outpatients by ownership, China, 2009–2017. Notes: Data source: China Health Statistical Yearbook 2010–2018. **A** proportions of public, private not-for-profit, and for-profit hospitals within China’s healthcare market; **B** the market share of hospital beds provided by different ownership types; **C** proportions of outpatients to seek medical services from public, private not-for-profit, and for-profit hospitals; **D** the market share of inpatient services provided by public, private not-for-profit, and for-profit hospitals. Note: The percentages do not add up to 100% for some years because of all the percentages are rounded to the nearest two decimal points

Many countries around the world have implemented a healthcare system composed of both public and private hospitals [1, 2], in which public hospitals, private not-for-profit hospitals, and private for-profit hospitals are expected to behave differently [3, 4]. Understanding the differences in the performance of hospitals by different ownership types is important to inform policy decisions made in the midst of health reforms. China initiated a systematic reform of its nationwide health system in 2009 [5, 6]. One of the key policies proposed in the reform was the lift of restrictions on private investments made in the healthcare sector [7]. This policy was primarily aimed at enhancing the responsiveness of the health system in meeting the increasingly diverse health care needs of the residents as well as facilitating improved healthcare quality and efficiency via encouraging competition within the hospital market [8, 9]. Since then, the number of for-profit and not-for-profit private hospitals grew at an unprecedented rate, with the total number of private hospitals exceeding that of public hospitals in 2015 (Fig. 1, percentage of public hospitals in total hospitals 47.37% vs percentage of private hospitals in total hospitals 52.63% in 2015).

There are long-standing debates over the performance of hospitals by different ownership types. Advocates for greater private sector participation argue that private for-profit hospitals are more cost-effective [10], more responsive to patients’ demands [11] and provide a higher quality of care [12, 13]. Given that the healthcare market has its unique characteristics [14], such as information asymmetry between providers and patients, criticism has been raised towards private for-profit hospitals for their stronger incentives to maximize their profits by inducing patients to use unnecessary services [15]. Under such context where patients have little information on health care quality, for-profit providers may choose to maximize their profits at the sacrifice of the quality of medical services delivered [16]. Compared with private for-profit hospitals, not-for-profit hospitals, including public and private not-for-profit hospitals might be more efficient as they have relatively lower administrative costs and are entitled to higher tax benefits. Unlike private for-profit hospitals, making profits is not set up as the primary goal for not-for-profit hospitals, which may facilitate the formation of a patient and society-friendly environment where physicians and managers tend to consider patients’ interests as the priority [4]. Those who highlight the public sector’s role in health care provision believe that public hospitals have a social welfare goal, which could lead to a more equitable system [4].

Quite a number of empirical studies explored the disparities in the quality of care and medical expenses among public, private not-for-profit, and for-profit hospitals, which provided inconsistent findings. For example, two reviews [17, 18] recently conducted among hospitals in Europe concluded that the evidence on the quality of care among different ownership types is too diverse to lead to a clear conclusion. As indicated by an earlier meta-analysis on eight relevant studies, five of those studies showed that private for-profit hospitals had higher payments for care (representing how much the healthcare provider received for the care delivered) than the private not-for-profit, while one study showed a totally opposite result [19]. A recent meta-analysis covering 21 studies found that public hospitals provided public health services at lower costs than the private sector [20].

Despite the growing number of studies on high-income countries [21, 22], evidence identified under the context of developing countries remains scant [23, 24]. Developing countries are typically confronted with greater constraints posed on their governmental budgets, which is a key financing source of healthcare expenses [25]. As the result, great efforts have been made by many developing countries to expand the role of the private sector in the health care delivery system. However, the capacities of healthcare financing in developing countries usually lag behind those of the developed countries and the business orientation of private hospitals in developing countries would be different from that in developed countries. Thus, in order to inform health policy, evidences are urgently needed to shed light upon the differences in the quality of care and medical expenses among hospitals with different ownership types under the context of developing countries.

Our study examines the disparities in health care quality and expenses across different hospital types in China. Public hospitals traditionally formed the backbone of China’s healthcare system [26]. Despite the growth of the private hospital sector over the last decade, the scales and sizes of private not-for-profit and for-profit hospitals remain smaller compared with public hospitals. In 2017, private not-for-profit and for-profit hospitals accounted for between 11 and 13% of total bed capacities (Fig. 1). In terms of service delivery, both types of private hospitals are similar, delivering between 7 and 9% of total outpatient and inpatient services in 2017, respectively. In China, the prices of health services in public hospitals are regulated by the government, and all public hospitals are covered by the social insurance payment program. Unlike the public hospitals, private hospitals are allowed to have two pricing options for medical services, namely those who choose to join the social insurance payment program must obey the pricing rules exactly the same as public hospitals, otherwise could set up their own prices for health services. Meanwhile, though the prices of health services in public hospitals or private hospitals which join the social insurance payment program are determined by the government, the process for treating and diagnosing diseases varied across hospitals, doctors, and patients [27]. Thus, the medical expenses and in-hospital mortality rates might be different across hospitals. Differences between the three types of hospitals are briefly described in Additional file 1: Table A1.

**Table 1:** Sample characteristics by hospital type in Sichuan province of China during the fourth quarters of 2016–2018

Variables	All	Public	Private not-for-profit	Private for-profit	*P* value^a^ (PNFP vs PFP vs PH)	*P* value^b^ (PNFP vs PH)	*P* value^b^ (PNP vs PH)
	(*N* = 64,171)	(*N* = 50,022)	(*N* = 8,597)	(*N* = 5,552)			
**Age, mean (SD)**	64.14 (17.06)	64.07 (17.02)	64.95 (17.34)	63.54 (16.92)	< 0.001	< 0.001	0.08
**Men, mean (SD)**	0.49 (0.50)	0.50 (0.50)	0.47 (0.50)	0.51 (0.50)	< 0.001	< 0.001	0.24
**Insurance type, mean (SD)**							
UEBMI	0.32 (0.47)	0.27 (0.45)	0.58 (0.49)	0.38 (0.49)	0.01	< 0.001	< 0.001
URBMI	0.27 (0.44)	0.28 (0.45)	0.19 (0.39)	0.26 (0.44)	< 0.001	< 0.001	< 0.001
NCMS	0.27 (0.45)	0.31 (0.46)	0.09 (0.28)	0.24 (0.43)	0.000	< 0.001	< 0.001
**Occupation, mean (SD)**							
Civil servant	0.05 (0.23)	0.06 (0.23)	0.04 (0.20)	0.04 (0.20)	< 0.001	< 0.001	< 0.001
Worker	0.03 (0.18)	0.03 (0.18)	0.03 (0.16)	0.02 (0.14)	< 0.001	< 0.001	< 0.001
Farmer	0.40 (0.49)	0.45 (0.50)	0.20 (0.40)	0.31 (0.46)	0.01	< 0.001	< 0.001
Freelancers	0.02 (0.14)	0.02 (0.14)	0.02 (0.13)	0.03 (0.18)	< 0.001	0.21	< 0.001
Unemployed	0.03 (0.17)	0.03 (0.16)	0.05 (0.21)	0.04 (0.19)	< 0.001	< 0.001	< 0.001
Retirement	0.11 (0.32)	0.08 (0.27)	0.30 (0.46)	0.13 (0.34)	0.01	< 0.001	< 0.001
**Charlson score, mean (SD)**	1.12 (1.37)	1.04 (1.32)	1.48 (1.56)	1.31 (1.43)	< 0.001	< 0.001	< 0.001
**Surgery, mean (SD)**	0.03 (0.18)	0.03 (0.18)	0.04 (0.19)	0.03 (0.18)	0.01	0.01	0.80
**LOS, mean (SD)**	9.62 (6.38)	9.38 (6.05)	10.75 (7.62)	9.98 (6.88)	< 0.001	< 0.001	< 0.001
**Admission type, mean (SD)**							
Emergency at arrival	0.10 (0.29)	0.10 (0.30)	0.08 (0.26)	0.06 (0.24)	< 0.001	< 0.001	< 0.001
Urgent at arrival	0.25 (0.44)	0.24 (0.43)	0.35 (0.48)	0.22 (0.41)	< 0.001	< 0.001	< 0.001
**Admission source, mean (SD)**							
Emergency admission	0.20 (0.40)	0.20 (0.40)	0.23 (0.42)	0.16 (0.37)	< 0.001	< 0.001	< 0.001
Outpatient admission	0.77 (0.42)	0.77 (0.42)	0.74 (0.44)	0.81 (0.39)	< 0.001	< 0.001	< 0.001

*Abbreviations*: *AMI* Acute myocardial infarction, *HF* Heart failure, *PFP* Private for-profit, *PH* Public hospital, *PNA* Pneumonia, *PNF* Private not-for-profit, *SD* Standard deviation, *UEBMI* Urban employee basic medical insurance, *URBMI* Urban resident basic medical insurance, *NCMS* New rural cooperative medical scheme

^a^*P* value was estimated by one-way ANOVA

^b^*P* value was estimated by paired t-test with Bonferroni adjustment

Only a handful of studies have examined the relationship between hospital ownership, quality of care and medical expenses in China. Eggleston (2010) [28] analyzed in-hospital mortality among public and private not-for-profit, and for-profit hospitals using hospital administrative data in Guangdong and found that there was no difference in in-hospital mortality for all diseases regardless of hospital ownership types. Xu (2015) [29] used survey data from the China’s Urban Resident Basic Medical Insurance Survey to analyze patients’ medical expense in public and private health care institutions and reported no difference in outpatient medical expenses between private and public healthcare institutions. Two other studies focused on self-reported patient satisfaction with the services received from private and public clinics and community health centers instead of hospitals [30, 31].

Our study is expected to bridge the gap embedded in the previous literature in several ways. First, our findings would shed light upon the differences in hospital quality and medical expenses among private for-profit and not-for-profit hospitals and public hospitals. The detailed patient-level information we retrieved from hospital administrative data enabled us to control for variations in quality and medical expenses arising from differences in patient case-mix and hospital characteristics using an extensive set of covariates. Second, existing studies predominantly used data prior to 2009, which predated the health care reforms that opened the hospital sector to private investments. As such, our adoption of more recent data from 2016 to 2018 would likely produce more meaningful outcomes reflective of the change in both public and private hospitals’ performances over the past decade in response to the implementation of policies since the initiation of the healthcare reform, especially in terms of the quality and expenses of medical services delivered by different types of hospitals.

## Data and methods

### Research area

Sichuan is one of the most densely populated provinces (82.62 million) in China, with a land area of 486,000 km^2^ and GDP per capita of 7,387 US dollars in 2018. From the geographical perspective, Sichuan province can be roughly seen as equally divided by Hengduan mountain into two sub-regions with distinctively different characteristics. The eastern half of the province is socioeconomically well-developed with dense population and large cities, while the western half is economically under-developed with sparse population and small towns. The vast variations in topography, population, and economy across the province, which to some degree is representative of the situation in many other parts of China, may explain the differences in healthcare resource allocation and growth in private hospital development across different regions within Sichuan (Additional file 1: Figure A1).

In China, hospitals are officially designated as primary (level 1), secondary (level 2), and tertiary (level 3) hospitals. The higher-level hospitals are positioned to deliver services for more critical patients. Generally, the higher-level hospitals are equipped with more hospital beds, better trained clinicians, and more advanced medical equipment. Specifically, the primary hospitals are usually equipped with less than 100 beds, as compared to the secondary hospitals with 100–500 beds, and the tertiary hospitals with more than 500 beds [32]. Similar to the nationwide situation from a holistic perspective, the private hospitals are relatively smaller in hospital size than the large public hospitals located within our study area of Sichuan province, regardless of the rapid development of the private hospital sector over the last decade. Until 2018, the private hospital beds in tertiary, secondary, and primary hospitals accounted for 4.3% (2,839), 62.4% (40,831), and 33.3% (21,769) of all hospital beds in Sichuan. However, the number of public hospitals decreased since 2009, mainly due to the fact that some primary public hospitals exited the market or upgraded to become higher-level hospitals (i.e., secondary or tertiary hospitals), and thus the number of secondary and tertiary public hospitals showed a slightly increase since 2009 [33].

### Data collection and sampling strategy

Our data was obtained from hospital discharge records provided by the Health Commission of Sichuan Province, China. The data comprised of all patients discharged from all Sichuan hospitals in the fourth quarters (October 1^st^ to December 31^th^) from 2016 to 2018. Hospital discharge record is a complete but highly condensed medical record for inpatients, which includes inpatients’ basic characteristics, diagnostic and treatment information, and medical expenses.

To assess the associations between hospital ownership types and in-hospital mortality and medical expenses, we selected three conditions which are the subject of many studies on quality of care [34, 35], namely acute myocardial infarction (AMI), heart failure (HF), and pneumonia (PNA). We focused on these three commonly diagnosed conditions to facilitate the comparison between our findings with those in the literature. These conditions were identified by the International Classification of Diseases 10th Revision (ICD-10), using the codes I21 and I22 for AMI, I50 for HF, and J12-J18 for PNA.

To improve comparability among different types of hospitals, we focused on patients discharged from secondary hospitals. As we mentioned above, there were only a few private tertiary level hospitals (only 4.3% of all private hospitals beds). Meanwhile, the primary hospitals are regarded to have no capacities to deal with severe medical conditions, especially those subsequently induced by AMI and HF which were chosen as the representative diseases in our study. Considering the trivial market share of private tertiary level hospitals, our research focused on secondary hospitals to ensure the comparability of the three types of hospitals investigated. The secondary hospitals, including private and public hospitals, usually have emergency departments to provide medical services for patients with critical conditions, such as AMI and HF.

A total of 64,171 patients (3,216 for AMI, 9,022 for HF, and 51,933 for PNA) were included in our analysis sample. Cases were chosen based on the following criteria: (1) patients with one of the three diseases previously selected; (2) patients were admitted to the secondary hospitals; (3) patients aged 18 years old and above; (4) patients with no erroneous information, and (5) patients with complete data on the variables we examined. Additional file 1: Figure A2 describes the sampling strategy. In our study, we simply deleted the missing data without using any imputation methods as only 22 participants were deleted due to missing data.

### Variables

The two key outcomes we analyzed were in-hospital mortality and the total medical expenses for the inpatient stay. In-hospital mortality is regarded as one of the most important hospital quality indicators and is usually used for measuring hospital quality [36]. The medical expenses retrieved from hospital discharge records included the total medical expenses, the out-of-pocket payment by the patients, and the reimbursements by the health insurance. The total expenses would be equal to the sum of the out-of-pocket and reimbursements. In our analysis, we used the total medical expenses from the perspective of the whole society. The key independent variable was hospital ownership. Following the literature [4, 37], hospital ownership was categorized into three categories: public, private not-for-profit, and private for-profit. In our analytical models, we followed the convention in the literature [38, 39] and adjusted for an extensive set of hospital-level and patient-level characteristics, as well as year dummies. The hospital characteristics included hospital type (general and non-general hospital), hospital grade (grade A, grade B, and non-graded), and hospital volume (total inpatient number of each condition). In China, quality standards of secondary hospitals are evaluated by the government, and hospitals are graded as grade A and B, with grade A indicating higher quality of care. Thus, we controlled for this quality grading in our analysis to some extent to ensure the comparability of hospitals with different ownerships.

Patient characteristics included age, gender, occupation (civil servants, workers, farmers, freelances, the unemployed, retirees, and others), insurance type (Urban Employee Basic Medical Insurance (UEBMI), Urban Resident Basic Medical Insurance (URBMI), New Cooperative Medical Scheme (NCMS), and others), surgery (yes and no), admission type (emergency, urgent, and elective at arrival), admission source (emergency admission, outpatient admission, and others), length of stay (LOS), and disease subtypes. We also included the Charlson comorbidity index (CCI)[40], which has been widely used as an indicator of patients’ comorbidities by previous studies under China’s context [41]. To derive the CCI, a total of 17 comorbidities were chosen with each comorbidity assigned a weighted score. The index is calculated as the sum of all scores to derive an indicator of disease burden (ranging from 0 to 37), with a higher score translating to a higher risk of death.

### Statistical analysis

To assess the associations between the hospital ownership and in-hospital mortality for PNA, HF, and AMI, separate multilevel logistic regressions were used for each disease. The dependent variable was a binary variable which was assigned the value 1 if the patient deceased in the hospital, and 0 otherwise:

1 $$Y_{ijt}\mathit\;\left|\mathit\;p_{ijt}\mathit\;\mathit=\right.\mathit\;bernoulli\left(p_{ijt}\right)$$

2 $$\log\left(\frac{p_{ijt}}{1-p_{ijt}}\right)=\beta_0+\delta ownership\_PFP_j+\psi ownership\_PNFP_j+\theta X_{ijt}+\kappa Z_{jt}+v_t+\gamma_j$$

3 $$\gamma_j\;\sim\;N\left(0,\sigma_1^2\right)$$

where *Y*_*ijt*_ denoted whether patient *i* in hospital *j* died during their hospital stay in *t* year. $p_{ijt}$ denoted the probability that patient *i* in hospital *j* died during their hospital stay in *t* year. $ownership\_PFP_{j}$ was a scalar of hospital ownerships for private for-profit. $ownership\_PNFP_{j}$ was a scalar of hospital ownerships for private not-for-profit. $X_{ijt}$ was a vector of patient-level control variables, including gender (male and female), age (continuous, time scale), insurance type (UEBMI, NCMS, URBMI, and others), occupation (civil servant, worker, farmer, freelances, unemployed, retirement, and others), Charlson score index (continuous), surgical condition (no and yes), admission type (emergency, urgent, and elective), admission source (emergency admission, outpatient admission, and others), LOS (continuous), and disease subtypes (dummies), and $Z_{jt}$ was a vector of hospital-level control variables (including hospital type, level, and volume). Further, $\delta$ was the parameter of interest to be estimated for private for-profit. $\psi$ was the parameter of interest to be estimated for private not-for-profit.$\beta_{0}$ was the constant term.$\theta$ was a vector of parameters for patient-level control variables while $\kappa$ was a vector of parameters for hospital-level control variables. Finally,$\nu_{t}$ was a set of year dummy variables,$\gamma_{j}$ a random effect at hospital-level with mean zero and variance $\sigma_{1}^{2}$. In order to deal with temporal changes of associations between hospital ownership types and in-hospital mortality, we additionally added an interaction term of year dummies and hospital ownership types in these models.

The multilevel linear regression was used to evaluate the association between hospital ownership types and medical expenses for PNA, HF, and AMI. As the medical expenses exhibited a substantial positive skewness, the logarithmic transformation was performed before the analyses:

4 $$\log\left(E_{ijt}\right)=\alpha_0+\eta ownership\_PFP_j+\varphi ownership\_PNFP_j+\tau X_{ijt}+\omega Z_{jt}+v_t+\gamma_j+\varepsilon_{ijt}$$

5 $$\gamma_j\sim N\left(0,\sigma_2^2\right);\varepsilon_{ijt}\sim N\left(0,\sigma^2\right)$$

where *E*_*ijt*_ denoted the medical expense of patient *i* in hospital *j* during their stay in hospitals in *t* year.$ownership\_PFP_{j}$ was a scalar of hospital ownerships for private for-profit. $ownership\_PNFP_{j}$ was a scalar of hospital ownerships for private not-for-profit. We used the same set of covariables as that for modeling mortality. $\eta$ was the parameter of interest to be estimated for private for-profit. $\varphi$ as the parameter of interest to be estimated for private not-for-profit.$\alpha_{0}$ was the constant term.$\tau$ was a vector of parameters for patient-level control variables while $\omega$ was a vector of parameters for hospital-level control variables. Finally,$\nu_{t}$ was a vector of dummy variable representing time specific factors,$\varepsilon_{ijt}$ was an error term, which has a mean of zero and a variance of $\sigma^{2}$.$\gamma_{j}$ a random effect at hospital-level with mean zero and variance $\sigma_{2}^{2}$. In order to deal with temporal changes of associations between ownership types and medical expenses, we additionally added an interaction term of year dummies and hospital ownership types in these models.

Likelihood ratio tests were further used to compare the difference of quality of care and medical expenses between the private not-for-profit hospitals and private for-profit hospitals.

Prior studies showed that in-hospital mortality varies by age and gender [39]. Considering the potential temporal changes of the associations between ownerships and in-hospital mortality and medical expenses, in a set of subgroup analyses, we investigated the presence of heterogeneous effects of hospital types by patients’ gender, age, insurance types, and year using likelihood ratio tests. To this end, we stratify our sample by gender (male and female), age (< 60 and ≥ 60 years), insurance types (UEBMI, NCMS, URBMI, and others), and year (2016, 2017, and 2018). We apply the same methods in all the subgroup analyses as with the main analyses.

In our study, we also performed the multilevel linear regression models to estimate the coefficient and its 95% CI for comparing the difference of LOS among public hospitals, private for-profit hospitals, and private not-for-profit hospitals. In a sensitivity analysis, to further reduce heterogeneity in the characteristics of patients visiting public and private hospitals, we applied coarsened exact matching (CEM) methods [42], to arrive at an analysis sample where private patients are comparable with public patients, which could partially reduce the selection bias. We match on gender, age, occupation, insurance types, ICC, surgical condition. To apply the CEM, hospital ownership was reclassified into two groups: public hospitals, and private hospitals with private not-for-profit and for-profit hospitals combined into one group. We used the estimated weights from the CEM to adjust the regression estimates from the multilevel logistic and linear regressions. The set of covariates used in these regressions is the same as the baseline analyses.

All statistical analyses were conducted with *R 4.2.0 in* our study, and all significance tests were two-sided with *P* < 0.05 being the level of statistical significance.

### Ethics consideration

We did not obtain ethics approval for this study, given that data are routinely collected by Health Commission. Besides, we did not use any private information of patients for analyses.

## Results

Table 1 summarizes our analysis sample. Our sample comprised of 64,171 inpatients from 548 hospitals, with 50,022 patients in public hospitals, 8,597 in private not-for-profit hospitals and 5,552 in private for-profit hospitals. Approximately half of our sample are male patients, with the mean age in private not-for-profit hospitals slightly older than public and private for-profit hospitals (65.0 vs 64.1 vs 63.5; *P* < 0.001). The proportion of patients with NCMS was the highest in public hospitals (31.0%), while patients with UEBMI made up the largest share by insurance types in private not-for-profit (58.5%) and for-profit hospitals (37.9%). Charlson score was higher in the private not-for-profit hospitals than public (1.5 vs 1.0; *P* < 0.001) and private for-profit hospitals (1.5 vs 1.3; *P* < 0.001). The characteristics of the sub-samples by disease (Additional file 1: Tables A2-4), on the whole, showed a similar pattern as that shown in Table 1. As for the hospital characteristics, the majority of public hospitals demonstrated to be grade A hospitals, followed by grade B, while the number of grade B and non-graded hospitals were close and predominant subtypes within private hospitals. Meanwhile, the average number of hospital beds, hospital medical equipment, nurses and doctors were higher in public hospitals than in private hospitals, which all reflected the larger scales that public hospitals have than private hospitals (Additional file 1: Figure A3 and Additional file 1: Table A5). Table 2 shows the observed in-hospital mortality rates and medical expenses for PNA, HF, and AMI. The mortality rates of all three conditions in public hospitals presented to be lower than those of private not-for-profit and for-profit hospitals, namely PNA (1.6% vs 3.3% vs 2.6%), HF (3.4% vs 5.8% vs 5.7%), and AMI (10.5% vs 20.4% vs 15.7%). In addition, pubic hospitals generally spent lower costs on the diseases investigated compared with private not-for-profit and for-profit hospitals [(PNA (5,634 vs 7,370 vs 6,692), HF (6,376 vs 7,921 vs 7,447), and AMI: 6,365 vs 8,494 vs 6,328)].

**Table 2:** In-hospital mortality rates and median medical expenses by conditions and hospital ownership types in Sichuan province of China during the fourth quarters of 2016–2018

Diseases	All	Public	Private not-for-profit	Private for-profit
**PNA**				
Cases	51,933	39,431	7,891	4,611
Observed rate (%)	1.96	1.62	3.29	2.56
**Cost**^**a**^	5,992.08 (6595.21)	5,634.48 (5899.94)	7,370.00 (7842.29)	6,691.99 (9064.85)
**HF**				
Cases	9,022	7,808	515	699
Observed rate (%)	3.69	3.37	5.83	5.72
**Cost**^**a**^	6,547.93 (5399.47)	6,376 (5101.76)	7,921 (7086.07)	7,447.12 (6794.77)
**AMI**				
Cases	3,216	2,783	191	242
Observed rate (%)	11.44	10.46	20.42	15.7
**Cost**^**a**^	6,488.35 (7884.31)	6,364.67 (7424.08)	8,493.95 (13,260.76)	6,327.72 (7140.79)

*Abbreviations*: *AMI* Acute myocardial infarction, *HF* Heart failure, *PNA* Pneumonia

^a^presented as mean (standard deviation)

Table 3 presents the regression estimates showing the degree of association between hospital ownership types and in-hospital mortality. We present the estimates for three different specifications, whereby hospital and patient characteristics were progressively included as regressors. We focus our discussion on the estimates from Model 3, which includes the full set of covariates. For AMI and HF, we found no difference in in-hospital mortality between public hospitals and private not-for-profit or for-profit hospitals, after controlling for an extensive set of patient and hospital characteristics. By contrast, PNA patients in private not-for-profit hospitals were 1.69 times (95% CI: 1.08 to 2.64) more likely to decease in hospitals compared with public hospitals. Similarly, patients in private for-profit hospitals were 1.67 times (95% CI: 1.06 to 2.62) more likely to decease than patients in public hospitals. Furthermore, the likelihood ratio tests were used to compare the differences between private not-for-profit and for-profit hospitals, which indicated that there was no difference in in-hospital mortality between private non-for-private hospitals and private not-for-profit hospitals for all three diseases (*p* = 0.956 for PNA; *p* = 0.240 for HF; *p* = 0.958 for AMI) (Additional file 1: Table A6).

**Table 3:** Associations between hospital ownership types and in-hospital mortality in Sichuan province of China during the fourth quarters of 2016–2018

Diseases	Dependent variable: In-hospital mortality		
	Model 1	Model 2	Model 3
	OR (95% CI)	OR (95% CI)	OR (95% CI)
**PNA**			
** Ownership**			
Public	Ref	Ref	Ref
Private not-for-profit	1.83 (1.18, 2.83)**	2.85 (1.78, 4.55)***	1.69 (1.08, 2.64)*
Private for-profit	1.44 (0.94, 2.23)	2.27 (1.41, 3.66)***	1.67 (1.06, 2.62)*
**Year**			
2016	Ref	Ref	Ref
2017	1.31 (1.09, 1.56)**	1.31 (1.10, 1.56)**	1.35 (1.10, 1.65)**
2018	1.60 (1.34, 1.93)***	1.60 (1.33, 1.92)**	1.42 (1.14, 1.77)**
**Random parts**			
Between-state variance	1.82	1.55	1.06
Intra Class Correlation (ICC)	0.36	0.32	0.24
N_Hospitals_	503	503	503
N_Individuals_	51,933	51,933	51,933
**HF**			
** Ownership**			
Public	Ref	Ref	Ref
Private not-for-profit	1.04 (0.58, 1.86)	1.02 (0.61, 1.75)	0.85 (0.46, 1.55)
Private for-profit	1.31 (0.77, 2.21)	1.34 (0.82, 2.20)	1.27 (0.72, 2.23)
**Year**			
2016	Ref	Ref	Ref
2017	1.06 (0.79, 1.43)	1.09 (0.81, 1.46)	1.07 (0.78, 1.49)
2018	0.77 (0.55, 1.08)	0.73 (0.53, 1.01)	0.57 (0.39, 0.83)**
**Random parts**			
Between-state variance	1.19	0.50	0.79
Intra Class Correlation (ICC)	0.27	0.13	0.19
N_Hospitals_	419	419	419
N_Individuals_	9,022	9,022	9,022
**AMI**			
** Ownership**			
Public	Ref	Ref	Ref
Private not-for-profit	2.11 (1.27, 3.54)**	1.69 (1.01, 2.83)*	1.17 (0.66, 2.09)
Private for-profit	1.56 (0.93, 2.62)	1.37 (0.81, 2.32)	1.15 (0.63, 2.09)
**Year**			
2016	Ref	Ref	Ref
2017	1.09 (0.82, 1.45)	1.12 (0.85, 1.49)	0.90 (0.65, 1.24)
2018	0.97 (0.71, 1.32)	0.91 (0.67, 1.24)	0.58 (0.40, 0.84)**
**Random parts**			
Between-state variance	0.77	0.47	0.58
Intra Class Correlation (ICC)	0.19	0.12	0.15
N_Hospitals_	352	352	352
N_Individuals_	3,216	3,216	3,216
**Control variables**			
Hospital characteristics^a^	No	Yes	Yes
Patient characteristics^b^	No	No	Yes

*Abbreviations*: *AMI* Acute myocardial infarction, *HF* Heart failure, *PNA* Pneumonia, *OR* Odds ratio, *CI* Confidence interval, *UEBMI* Urban employee basic medical insurance, *URBMI* Urban resident basic medical insurance, *NCMS* New rural cooperative medical scheme, *Ref* Reference category

^a^Hospital characteristics included hospital type (general and non-general), hospital grade (grade A, grade B and non-graded), and volume (continuous, log-transform)

^b^Patient characteristics included gender (male and female), age (continuous, time scale), insurance type (UEBMI, NCMS, URBMI, and others), occupation (civil servant, worker, farmer, freelances, unemployed, retirement, and others), Charlson score index (continuous), surgical condition (no and yes), admission type (emergency, urgent, and elective), admission source (emergency admission, outpatient admission, and others), LOS (continuous, log-transform), and disease subtypes (dummies)

^*^Indicating 0.01 ≤ *p*-value < 0.05

^**^Indicating 0.001 ≤ *p*-value < 0.01

^***^Indicating *p*-value < 0.001

In addition, we found the in-hospital mortality increased in patients with PNA while decreased in patients with HF and AMI across years. Additional file 1: Table A8 showed the results when we added the interaction term of ownership types and year dummies in the models, and suggested none of the interaction terms posed interactive effects on in-hospital mortality for these three conditions using Wald tests (*P* for interaction ≥ 0.54; Additional file 1: Table A7). Similar results were reported using likelihood ratio tests, suggesting little heterogeneities existed in the relationship between hospital ownership and in-hospital mortality for three conditions across years (*P* for interaction ≥ 0.52; Additional file 1: Table A10).

We now turn to look at the relationship between hospital ownership types and medical expenses. The estimates are presented in Table 4. For AMI, we found that medical expenses did not significantly differ across hospital ownership types. For HF, our estimates indicated that medical expenses in private not-for-profit hospitals were higher than that in public hospitals. Specifically, mean medical expenses for PNA were 9% higher in private not-for-profit compared with public hospitals, while no significant differences were found between private for-profit hospitals and public hospitals in this aspect. For PNA, mean medical expenses were 10% higher in private not-for-profit hospitals, and 11% higher in for-profit hospitals compared with public hospitals. Furthermore, the likelihood ratio tests were used to compare the differences between private not-for-profit and for-profit hospitals. No differences were found between those two types of private hospitals in medical expenses for three diseases (*p* = 0.764 for PNA; *p* = 0.270 for HF; *p* = 0.543 for AMI) (Additional file 1: Table A8).

**Table 4:** Associations between hospital ownership types and medical expenses in Sichuan province of China during the fourth quarters of 2016–2018

Diseases	Model 1	Model 2	Model 3
	coef (95% CI)	coef (95% CI)	coef (95% CI)
**PNA**			
** Ownership**			
Public	Ref	Ref	Ref
Private not-for-profit	0.09 (0.02, 0.15)**	0.16 (0.09, 0.23)***	0.10 (0.06, 0.15)***
Private for-profit	0.06 (0.00, 0.12)*	0.13 (0.07, 0.19)***	0.11 (0.07, 0.15)***
**Year**			
2016	Ref	Ref	Ref
2017	-0.02 (-0.03, -0.01)**	-0.02 (-0.03, -0.01)**	-0.01 (-0.02, 0.01)
2018	0.08 (0.06, 0.09)***	0.07 (0.06, 0.08)***	0.04 (0.03, 0.05)***
**Random parts**			
Between-state variance	0.13	0.10	0.05
Intra Class Correlation (ICC)	0.28	0.24	0.30
N_Hospitals_	503	503	503
N_Individuals_	51,933	51,933	51,933
**HF**			
** Ownership**			
Public	Ref	Ref	Ref
Private not-for-profit	0.11 (0.01, 0.22)*	0.24 (0.13, 0.36)***	0.09 (0.01, 0.17)*
Private for-profit	0.05 (-0.05, 0.14)	0.15 (0.05, 0.26)**	0.04 (-0.03, 0.11)
**Year**			
2016	Ref	Ref	Ref
2017	0.00 (-0.03, 0.04)	0.00 (-0.03, 0.03)	0.01 (-0.02, 0.02)
2019	0.07 (0.03, 0.11)***	0.07 (0.03, 0.10)***	0.02 (-0.01, 0.05)
**Random parts**			
Between-state variance	0.11	0.10	0.06
Intra Class Correlation (ICC)	0.23	0.21	0.30
N_Hospitals_	419	419	419
N_Individuals_	9,022	9,022	9,022
**AMI**			
** Ownership**			
Public	Ref	Ref	Ref
Private not-for-profit	-0.03 (-0.24, 0.18)	0.20 (-0.01, 0.42)	0.05 (-0.07, 0.18)
Private for-profit	0.01 (-0.19, 0.21)	0.19 (-0.23, 0.40)	0.01 (-0.11, 0.13)
**Year**			
2016	Ref	Ref	Ref
2017	-0.01 (-0.09, 0.07)	-0.01 (-0.09, 0.07)	0.04 (-0.01, 0.08)
2018	0.07 (-0.02, 0.16)	0.09 (0.01, 0.18)*	0.06 (0.01, 0.11)*
**Random parts**			
Between-state variance	0.22	0.17	0.06
Intra Class Correlation (ICC)	0.21	0.17	0.20
N_Hospitals_	352	352	352
N_Individuals_	3,216	3,216	3,216
**Control variables**			
Hospital characteristics^a^	No	Yes	Yes
Patient characteristics^b^	No	No	Yes

*Abbreviations*: *AMI* Acute myocardial infarction, *HF* Heart failure, *PNA* Pneumonia, *Coef* Coefficient, *CI* Confidence interval, *UEBMI* Urban employee basic medical insurance, *URBMI* Urban resident basic medical insurance, *NCMS* New rural cooperative medical scheme, *Ref* Reference category

^a^Hospital characteristics included hospital type (general and non-general), hospital grade (grade A, grade B and non-graded), and volume (continuous, log-transform)

^b^Patient characteristics included gender (male and female), age (continuous, time scale), insurance type (UEBMI, NCMS, URBMI, and others), occupation (civil servant, worker, farmer, freelances, unemployed, retirement, and others), Charlson score index (continuous), surgical condition (no and yes), admission type (emergency, urgent, and elective), admission source (emergency admission, outpatient admission, and others), LOS (continuous, log-transform), death (no and yes), and disease subtypes (dummies)

^*^Indicating 0.01 ≤ *p*-value < 0.05

^**^Indicating 0.001 ≤ *p*-value < 0.01

^***^Indicating *p*-value < 0.001

In addition, medical expenses associated with PNA were found to increase for patients across years. Additional file 1: Table A9 showed the results when we added the interaction term of ownership types and year dummies in the models, and suggested none of the interaction terms posed interactive effects on medical expenses for HF and AMI using Wald tests (*P* for interaction ≥ 0.13). Nevertheless, both ownership and year were found to have posed significantly positive interactive effects on medical expenses for PNA (*P* for interaction < 0.001; Additional file 1: Table A9), suggesting that mean medical expenses increased higher in private not-for-profit hospitals, and for-profit hospitals compared with public hospitals across years. Similar results were reported using likelihood ratio tests, suggesting the existence of heterogeneities in the relationship between hospital ownership and medical expenses for PNA across years (*P* for interaction < 0.001; Additional file 1: Table A11).

Additional file 1: Table A10-A11 show the results for subgroup analyses and indicate little heterogeneities existed in the relationship between hospital ownership and in-hospital mortality or medical expenses across age, genders, insurance types, and year, except for age (*P* for interaction < 0.001), insurance type (*P* for interaction < 0.001) and year (*P* for interaction < 0.001) in PNA for medical expenses.

The results listed in Additional file 1: Table A12-A13 show the associations between hospital ownership and in-hospital mortality and medical expenses, respectively. Specifically, private hospitals showed higher in-hospital mortality than public hospitals for PNA but no difference for AMI and HF. In addition, no differences were found between the for-profit and not-for-profit hospitals in terms of in-hospital mortality and medical expense for all diseases, both prior to and after matching. As for the medical expense, private hospitals had higher medical expenses than public hospitals for PNA while there were no significant differences between the private and public hospitals for AMI. Meanwhile, no differences were found between the for-profit and not-for-profit hospitals in terms of in-hospital mortality and medical expense for AMI, and PNA, both prior to and after matching. However, a slight difference was found in HF before and after matching in these two aspects.

Additional file 1: Table A14 shows the results for the associations between hospital ownership and LOS for three conditions. No significant associations between hospital ownership and LOS were found among inpatients with AMI and PNA. But we found that private not-for profit hospitals presented to have stronger associations with increased LOS among inpatients with HF, compared with public hospitals.

## Discussion

Since the government alleviated the constraints previously posed on the private investments in China’s healthcare system in 2009, the private hospitals have grown substantially across the nation. In our study, we chose three commonly diagnosed and high-impact diseases (PNA, HF, and AMI) to investigate the association of hospital ownership types with in-hospital mortality and medical expenses. We found that private hospitals, regardless of not-for-profit or for-profit in nature, showed significantly higher in-hospital mortality for PNA than public hospitals, but differences were not found for AMI or HF in this aspect. Meanwhile, private hospitals also exhibited higher medical expenses for HF (but not for private for-profit) and PNA, but not for AMI than public hospitals. Moreover, no differences were observed between the private not-for-profit and for-profit hospitals both in terms of in-hospital mortality and medical expenses for all three diseases selected for analysis.

Unlike previous studies which reported that the private hospitals were more likely to admit patients with less severe symptoms and conditions [21], the Charlson score of patients was found to be the highest in private not-for-profit, followed by private for-profit and public hospitals, with the higher Charlson score to some extent indicating higher severity of medical conditions. Meanwhile, our results added new evidences into relevant studies that attempted to investigate such issue under China’s context, which produced slightly different findings. For example, in one previous study that performed a multi-stage design through 2004 to 2005 to collect data from a sample composed of governmentally-owned and private hospitals in Guangdong Province, a southern province in China, no differences were identified in in-hospital mortality for all diseases investigated among public, private not-for-profit, and for-profit hospitals after controlling for hospital-level potential confounders [28]. In another study which retrieved self-reported outpatient expenses over the past 2 weeks from China's Urban Resident Basic Medical Insurance Survey from 2008 to 2010, and used the variable to roughly represent outpatient medical expenses, no differences were observed in this aspect between private and public healthcare institutions [29]. These inconsistent outcomes might have been induced due to the adoption of different methods, study regions and target population groups. Instead of looking at the in-hospital mortality rates or total outpatients’ expenses reported at the hospital level, we managed to achieve better control of patients’ case-mix via conducting more in-depth investigations into the in-hospital mortality and inpatients expenses associated with those three commonly diagnosed diseases. As suggested by previous research that patients’ age, severity of medical conditions, racial disparities and comorbidities were associated with in-hospital mortality [43, 44], our patient-level investigation based on three commonly diagnosed diseases admitted for in-patient services has great potential to reduce the inherent heterogeneities embedded in both patients and diseases as well as to add new evidences into the existing literature.

Globally, evidences to reveal the associations between hospital ownership types and hospital quality and medical expenses were controversial. For example, a meta-analysis pooling 15 studies [4] (including both primary studies and meta-analysis studies) found higher mortality and medical expenses in private for-profit institutes than private not-for-profit facilities in high-income countries, while reported no clear differences in mortality or expenses between the not-for-profit providers and public ones in both high-income and middle- and low-income countries. Two meta-analysis conducted in US found that private for-profit hospitals increased the risk of death [45] and had higher expenses than the private not-for-profit ones [19]. Meanwhile, the evidences [17, 18] from European countries reported that the disparities in the quality of care among different hospital ownership types failed to produce a clear conclusion. Looking at more recently published articles, a meta-regression which pooled 21 studies from six countries or cities (including USA, Belgium, Germany, Spain, Italy, and Taiwan) indicated that public hospitals may provide public health services at cheaper prices than the private sectors [20]. Another systematic analysis focusing on the middle- and low-income countries suggested that diagnostic accuracy and adherence to medical management standards were worse among private sector care providers than the public ones [3]. According to the findings provided by relevant studies from the current literature the results seemed to vary across countries and also showed some differences compared with our results. Such inconsistencies might have been induced by different contexts where different organizations, financing and regulations of healthcare system might also affect the outcomes. The adoption of different methods or outcome indicators is another factor potentially associated with outcome disparities. Unlike the US where the private not-for-profit hospitals dominated in the healthcare system, the development of private hospitals in China is still at an early stage. Meanwhile, the financing capacities of healthcare systems implemented in different countries might also result in the differences in hospitals’ behaviors in response to region-specific contexts. Our research added new evidences for middle- and low- income countries and indicated that the public health care providers delivered at least equal or even better quality of medical services at lower medical expenses than the private ones, while no differences were observed between private not-for-profit and private for-profit hospitals in this aspect.

The unevenly distributed healthcare professionals across different hospital ownership types could partly explain the disparities in the in-hospital mortality. Despite the rapid expansion of both private not-for-profit and for-profit hospitals over the last decade, the development of the newly emerged hospitals were still at an immature stage where a relatively poorly-established human resource management system is not capable of ensuring the allocation of adequate medical professionals within the healthcare organization who are highly-skilled and experienced enough to guarantee the quality of care as in public hospitals [46]. Meanwhile, both private not-for-profit and for-profit hospitals have been confronted with bad reputations they previously gained from the occurrence of severe adverse medical events that were exposed to the public to escalate safety concerns [47]. All these issues posed huge obstacles for the private not-for-profit and for-profit hospitals in forming a team of well-qualified doctors and nurses towards enhanced quality of medical services delivered. Moreover, there are explicit clinical guidelines and consensus for the treatments of AMI and HF [48, 49], but not for PNA [50]. As PNA treatment tends to rely more on the doctors’ competence and experiences than AMI and HF, it is not difficult to predict that the lack of qualified healthcare professionals likely leads to higher mortality of PNA in both private not-for-profit and for-profit hospitals than the public ones, thus resulting in the heterogenous relationships among different diseases as suggested by our findings. It should also be noted that despite no essential temporal heterogeneities were found in the relationship between hospital ownership types and in-hospital mortality for all three conditions investigated across years, the in-hospital mortality of PNA did demonstrate notable increase over the corresponding study period. This suggested the necessity of developing explicit clinical guidelines to arrive at widely accepted expert consensus to facilitate the treatment and diagnostic procedures of PNA, as well as for other similar conditions.

As the fee-for-service payment method prevails in China’s nationwide healthcare system, healthcare providers are given unprecedentedly attractive financial incentives from over-treatment [51]. Under such context, the private for-profit hospitals, as profit-oriented facilities, may have more motivations to lure their customers to pay for unnecessary services as a tactic to maximize their profits, thus leading to higher medical expenses than public hospitals. Though private not-for-profit hospitals in China are never expected to financially behave like the private for-profit ones, the prior literature [52] indicated that the private not-for-profit hospitals were still on their way towards profit-oriented goals, which would be quite different from the cases in developed countries, such as the US [19]. Such evidences found in the previous literature were consistent with our findings that both private not-for-profit and for-profit hospitals had higher medical expenses than in the public hospitals for PNA, and higher medical expenses in private-not-for-profit than in the public hospitals for HF, while there were no statistical differences found between them. Meanwhile, we found a temporal heterogeneity in the relationship between hospital ownership and medical expenses across these three years for PNA, and the mean medical expenses of PNA increased higher in private not-for-profit hospitals, as well as in for-profit hospitals compared with public hospitals across years. This gap of medical expenses embedded between public hospitals and private hospitals increased over years, which might be partially explained by the unique characteristics of private hospitals as profit-gaining institutes in nature. However, as our study merely focused on a three-year time span, whether such change would persist as a long-term trend remain unclear based on this time point of analysis. As such, the disparities embedded in medical expenses across hospital ownership types should be constantly examined as a critical issue to produce more meaningful implications from a long-term perspective.

### Policy implications

According to our research, three major policy recommendations are provided. First, a well-established public reporting system is rather essential to facilitate constant surveillance over the quality of care and medical expenses among a wide range of healthcare institutes. Specifically, it is noteworthy that despite public hospitals are capable of delivering medical services with better quality and lower prices compared with the private ones, a large number of patients still chose to seek healthcare services from private hospitals. This implies that private hospitals were able to attract their patients by improving non-clinical quality of care which could be directly perceived by patients, instead of hard-to-monitor clinical quality of care. This proves the necessity of establishing a public reporting system as an effective solution to mitigating such information asymmetry embedded between healthcare providers and receivers. By doing so, competition within healthcare markets would likely be intensified among provides, thus further facilitating the constant improvement of healthcare quality as well as curbing the medical expense inflation for both public and private hospitals. As we mentioned earlier, medical conditions that currently lack explicit clinical guidelines on their treatment and diagnostic procedures should be set up as a high-risk group to be frequently reported in the system under top-level surveillance.

Second, enhancing the governance capabilities of private not-for-profit hospitals is rather critical. As suggested by our findings, no differences were found between the private not-for-profit and private for-profit hospitals both in terms of both healthcare quality and medical expenses. This should be recognized as an undesired outcome against the governmental intention to build these two subtypes of private hospitals, which expects that the for-profit hospitals should strive towards profit-oriented goals, while the not-for-profit ones should aim at maximizing the benefits of the whole society or patients instead of gaining maximum profits. Under the current challenging climate where all types of private hospitals are striving to survive in the market, how to guide those hospitals back to the track initially set up by the government towards improved quality of care at lower prices is predicted to be the major challenge down the road.

Third, our findings also provide meaningful implications to inform other nations confronted with similar challenges. China's nationwide healthcare system experienced a huge transition from public hospital-dominated system to highly mixed-ownership system since 2009. Despite rapid expansion over the past decades, private hospitals still have a long way to go before achieving the expected goals initially set up at the governmental level, due to the fact that private hospitals still lack behind the public ones both in the quality of medical services delivered and the medical expenses induced. This suggested that reform-related strategies should be tailored for the unique attributes of healthcare markets under different contexts, in addition to healthcare outcome disparities potentially induced by unevenly distributed healthcare professionals among different institutional types, or other context-specific factors such as bad reputation previously gained from undesired performances that result in the escalation of safety concerns. Under the current climate where COVID-19 has posed unprecedented challenges for current healthcare system, the development of private hospitals as a long-term battle towards expected goals is foreseeable.

### Limitations

There are several limitations in our study. First, despite that the in-hospital mortality of those three commonly diagnosed diseases has been frequently used by worldwide studies to assess the quality of care for hospitals, the comprehensiveness of using this indicator to reflect the overall quality of care remains limited. Second, some confounding factors could not be controlled due to the limitation of data or could not be identified due to the lack of theoretical evidences, such as the exact time points that hospitals were established or the details throughout treatment procedures, which might be a con-founder to affect the actual quality of care. Meanwhile, it was also hard to differentiate some confounding factors and mediators. As we have discussed before, the varied quality of care for hospitals by different ownership types might be partially explained by unevenly distributed medical talents who had been attracted to work in different hospitals, which might serve as a mediator in our research. However, such factor failed to be added as a confounding factor to further examine whether or not such disparities found among different hospital ownership types were mainly induced by hospitals' reputations they previously gained from the public due to data availability issue. Third, as our research only included the secondary hospitals from Sichuan provinces into our final analysis, the results from our study might not be potent enough to be generalized to the entire healthcare market in China. Forth, as public hospitals might have also been improved in efficiency along with the rapid development of private hospitals settings, the assumption that hospitals’ performances remained constant over our study period might not be reasonable enough for comparison. As we were not able to construct a panel data at the patient level due to data limitations, more panel studies are still needed to better examine the performance disparities between private and public hospitals after considering longitudinal changes in private and public hospitals. In addition, potential selection bias was inevitable given different characteristics inherent in patients along with the severity of medical conditions across three types of hospitals. As an attempt to avoid these selections bias, we adjusted for several potential patients’ characteristics in main analyses, and used coarsened exact matching method to arrive at an analysis sample where private patients are comparable with public patients in sensitive analyses. Last but not least, our datasets only included the fourth quarters of the year because of the availability of data. This inevitably added to some potential selection bias due to constantly changing patterns over seasons. In addition, the 3-year span we analyzed in this study was not long enough to reflect the long-term trends of disparities embedded in quality of care and medical expenses among hospitals with different ownership types. As such, more comprehensive data collected throughout the entire year range should be adopted in future studies to warrant our findings.

## Conclusions

The public hospitals were able to deliver healthcare with equal or better quality at lower medical expenses than private not-for-profit and for-profit hospitals, while no differences were found between private not-for-profit and for-profit hospitals both in the quality of care and medical expenses in China.

## Supplementary Information

**Additional file 1:** **Figure A1.** Geographic position, topography,economic development, and population of Sichuan Province, China, 2017. **FigureA2.** The flow chart for sampling procedure in our study. **Figure A3.** The basic characteristics of hospitals by ownership in Sichuan province of China during the fourth quarters of 2016-2018. (A) Hospital bed number; (B) Hospital medical equipment number; (C) hospital nurse number; (D) hospital doctor number. **Table A1.** The characteristics of the different hospitals by ownership. **Table A2.** The characteristics for PNA in Sichuan province of China during the fourth quarters of 2016-2018. **Table A3.** The characteristics for HF in Sichuan province of China during the fourth quarters of 2016-2018. **Table A4.** The characteristics for AMI in Sichuan province of China during the fourth quarters of 2016-2018. **Table A5. **The basic characteristics of hospitals by ownership in Sichuan province of China during the fourth quarters of 2016-2018. **Table A6.** The difference of in-hospital mortality among public, private not-for-profit, and private for-profit hospitals in Sichuan province of China during the fourth quarters of 2016-2018. **Table A7.** The relationship between the interaction of hospital ownership types and year with in-hospital mortality rate in Sichuan province of China during the fourth quarters of 2016-2018. **Table A8.** The difference of medical expenses among public, private not-for-profit, and private for-profit hospitals in Sichuan province of China during the fourth quarters of 2016-2018. **Table A9.** The relationship between the interaction of hospital ownership and year with medical expenses in Sichuan province of China during the fourth quarters of 2016-2018. **Table A10.** The subgroup analyses for the relationship between hospital ownership types and in-hospital mortality in Sichuan province of China during the fourth quarters of 2016-2018. **Table A11.** The subgroup analyses for the relationship between the hospital ownership and medical expense in Sichuan province of China during the fourth quarters of 2016-2018. **Table A12. **The relationship between whether private or not or whether for-profit or not and in-hospital mortality in Sichuan province of China during the fourth quarters of 2016-2018. **Table A13.** The relationship between whether private or not or whether for-profit or not and medical expenses in Sichuan province of China during the fourth quarters of 2016-2018. **Table A14.** Associations between hospital ownership types and LOS in Sichuan province of China during the fourth quarters of 2016-2018.

## Data Availability

The data that support the findings of this study are available from the Health Commission of Sichuan Province but restrictions apply to the availability of these data, which were used under license for the current study, and so are not publicly available. Data are however available from the authors upon reasonable request and with permission of the Health Commission of Sichuan Province, China.

## Abbreviations

AMI: Acute Myocardial Infarction
CCI: Charlson Comorbidity Index
CI: Confident Interval
COEF: Coefficient
HF: Heart Failure
LOS: Length of Stay
OR: Odds Ratio
PFP: Private For-Profit
PH: Public Hospital
PNA: Pneumonia
PNF: Private Not-For-Profit
UEBMI: Urban Employee Basic Medical Insurance
URBMI: Urban Resident Basic Medical Insurance
NCMS: New Rural Cooperative Medical Scheme
REF: Reference Category
SD: Standard Deviation
