# Supplementary material for: The relationship between hospital ownership, in-hospital mortality, and medical expenses: an analysis of three common conditions in China
Source: Arch Public Health. 2023 Feb 10;81:19. doi: 10.1186/s13690-023-01029-y (PMC9911958; doi:10.1186/s13690-023-01029-y)
Supplement: Supplementary file 1 — Additional file 1: Figure A1. Geographic position, topography,economic development, and population of Sichuan Province, China, 2017. FigureA2. The flow chart for sampling procedure in our study. Figure A3. The basic characteristics of hospitals by ownership in Sichuan province of China during the fourth quarters of 2016-2018. (A) Hospital bed number; (B) Hospital medical equipment number; (C) hospital nurse number; (D) hospital doctor number. Table A1. The characteristics of the different hospitals by ownership. Table A2. The characteristics for PNA in Sichuan province of China during the fourth quarters of 2016-2018. Table A3. The characteristics for HF in Sichuan province of China during the fourth quarters of 2016-2018. Table A4. The characteristics for AMI in Sichuan province of China during the fourth quarters of 2016-2018. Table A5. The basic characteristics of hospitals by ownership in Sichuan province of China during the fourth quarters of 2016-2018. Table A6. The difference of in-hospital mortality among public, private not-for-profit, and private for-profit hospitals in Sichuan province of China during the fourth quarters of 2016-2018. Table A7. The relationship between the interaction of hospital ownership types and year with in-hospital mortality rate in Sichuan province of China during the fourth quarters of 2016-2018. Table A8. The difference of medical expenses among public, private not-for-profit, and private for-profit hospitals in Sichuan province of China during the fourth quarters of 2016-2018. Table A9. The relationship between the interaction of hospital ownership and year with medical expenses in Sichuan province of China during the fourth quarters of 2016-2018. Table A10. The subgroup analyses for the relationship between hospital ownership types and in-hospital mortality in Sichuan province of China during the fourth quarters of 2016-2018. Table A11. The subgroup analyses for the relationship between the hospital ownership and medical expens [file 13690_2023_1029_MOESM1_ESM.docx]

Additional file 1


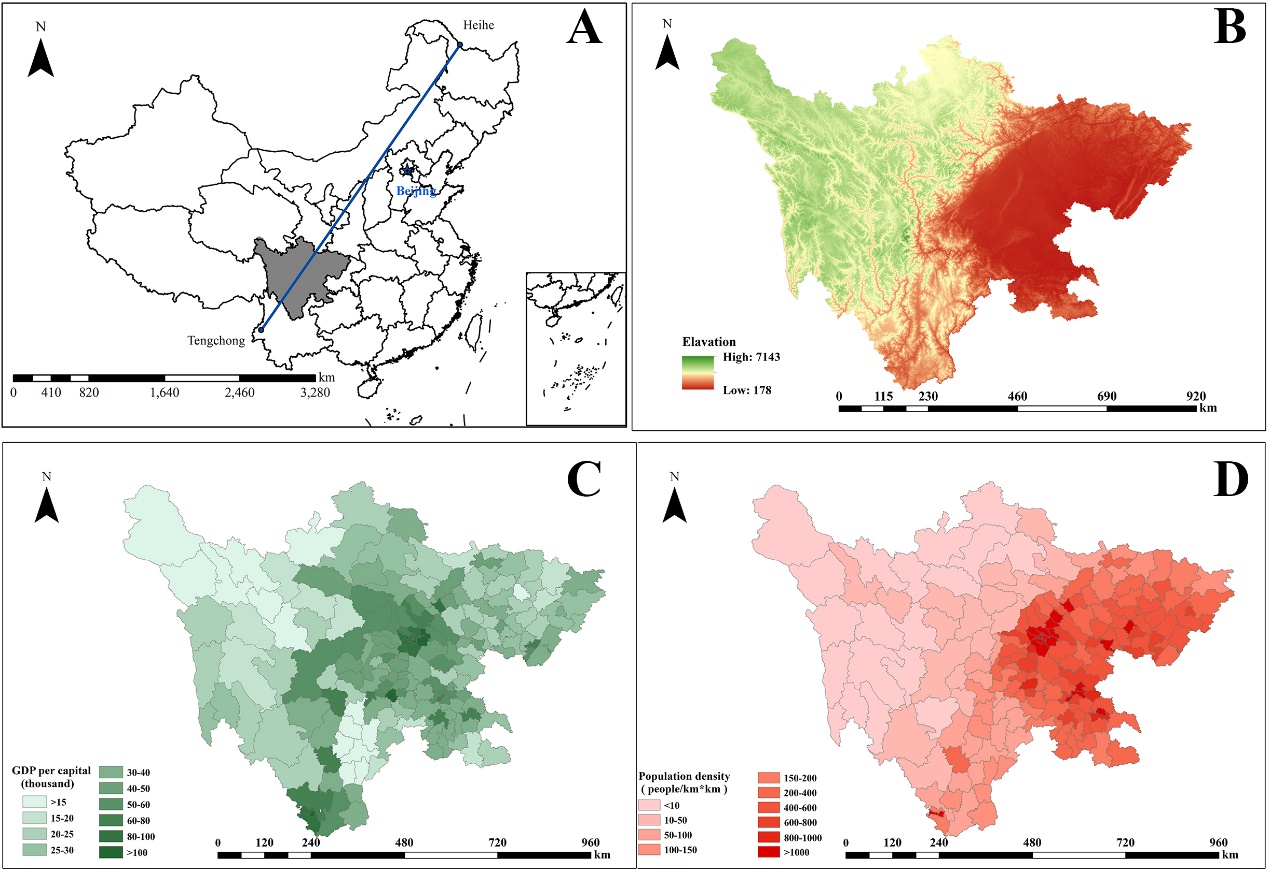


Figure A1. Geographic position, topography, economic development, and population of Sichuan Province, China, 2017.

(A) geographic position of Sichuan; (B) topography of Sichuan; (C) GDP per capital among counties in Sichuan; (D) population density among counties in Sichuan

**Inpatients were admitted to hospitals with AMI, HF or PNA**

**n= 512,401 (AMI: 18,752; HF: 24,432; PNA: 469,217)**

**Exclusion:**

**Not treated by secondary hospitals (n=325,995)**

**Lacking necessary characteristic information (n=** **22)**

**Aged less than 18 years old (n= 121,990)**

**with extreme or unreasonable information (n=131)**

**The final sample sizes**

**n= 64,263 (AMI: 3,216; HF: 9,022; PNA: 51,933)**

Figure A2. The flow chart for sampling procedure in our study.

Abbreviations: AMI, acute myocardial infarction; HF, heart failure; PNA, pneumonia.

**
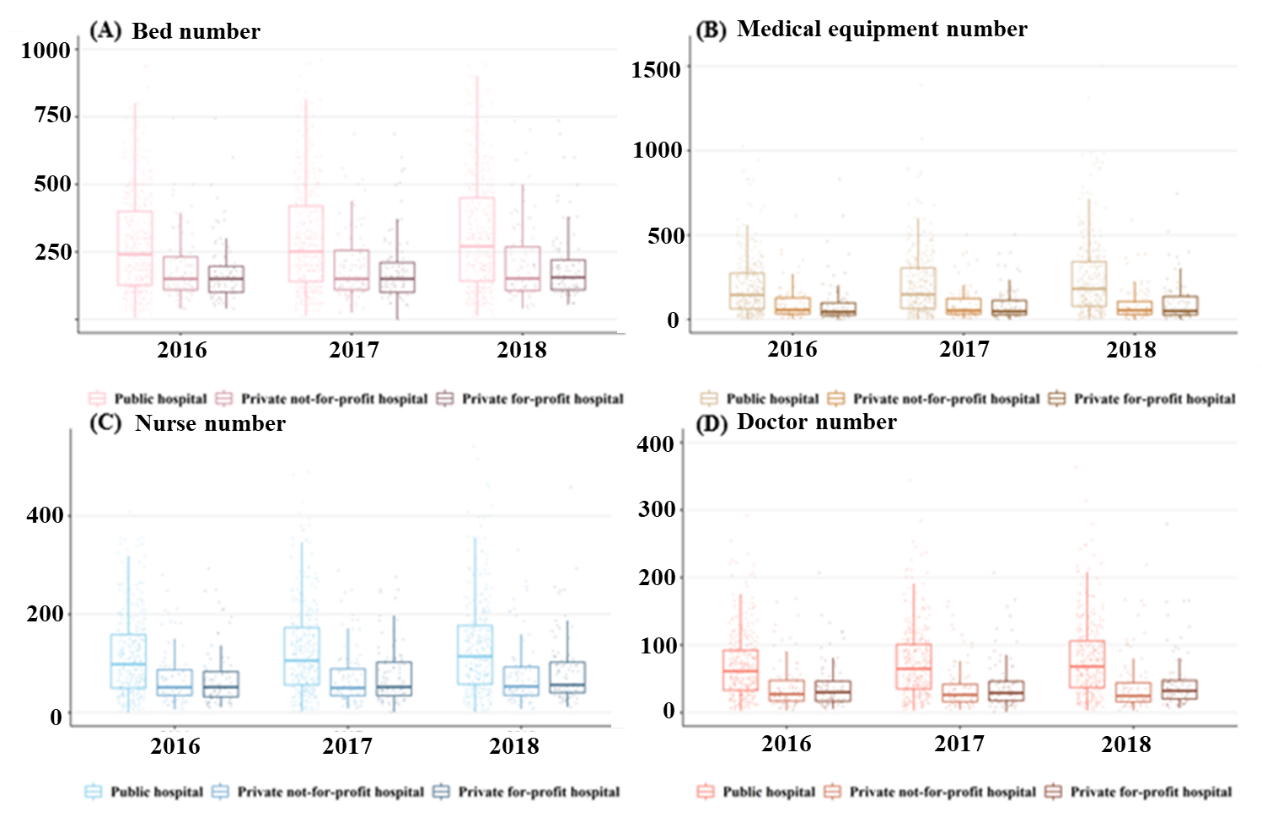
**

Figure A3. The basic characteristics of hospitals by ownership in Sichuan province of China during the fourth quarters of 2016-2018.

(A) Hospital bed number; (B) Hospital medical equipment number; (C) hospital nurse number; (D) hospital doctor number.

Table A1. The characteristics of the different hospitals by ownership.

| Characteristics | Public hospital | Private not-for-profit hospital | Private for-profit hospital |
| --- | --- | --- | --- |
| Owner of hospitals | Government owned | Not government owned | Not government owned |
| Services types | Provide basic medical services; Complete other tasks assigned by the government; Provide a small amount of non-essential services | Encourage to provide services which are undersupplied by public hospitals | Determine medical services according to market demand; Encourage to provide services which are undersupplied by public hospitals |
| Operational goals | Maximizing the benefit of society or patients | Maximizing the benefit of society or patients | Profit-oriented |
| Right of surplus distribution | Cannot distribute surplus and surplus can only be used for further development | Cannot distribute surplus to those who control the organization and surplus can only be used for further development | Can distribute all profits (net revenues less expenses) to owners |
| Tax treatment—personal/corporate income tax | No tax applicable | Exempted from corporate tax if they comply with price set by the state for medical services | Exempted from corporate tax for three years, and after that, owners of hospitals have to pay personal income tax from earnings and corporate tax |
| Government subsidies | Yes | No | No |

Table A2. The characteristics for PNA in Sichuan province of China during the fourth quarters of 2016-2018.

| **Variables** | All | Public | Private not-for-profit | Private for-profit | *P* for all^a^ | *P* (PNFP vs PH)^b^ | *P* (PNP vs PH)^b^ |
| --- | --- | --- | --- | --- | --- | --- | --- |
|  | (*N*=51,933) | (*N*=39,431) | (*N*=7,891) | (*N*=4,611) |  |  |  |
| **Age, mean (SD)** | 62.33 (17.55) | 61.97 (17.60) | 64.16 (17.49) | 62.30 (17.07) | <0.001 | < 0.001 | 0.681 |
| **Men, mean (SD)** | 0.49 (0.50) | 0.49 (0.50) | 0.46 (0.50) | 0.51 (0.50) | <0.001 | < 0.001 | 0.180 |
| **Insurance type, mean (SD)** |  |  |  |  |  |  |  |
| UEBMI | 0.34 (0.48) | 0.29 (0.45) | 0.60 (0.49) | 0.39 (0.49) | 0.000 | < 0.001 | < 0.001 |
| URBMI | 0.25 (0.43) | 0.27 (0.44) | 0.17 (0.38) | 0.25 (0.43) | <0.001 | < 0.001 | 0.001 |
| NCMS | 0.27 (0.45) | 0.31 (0.46) | 0.08 (0.27) | 0.26 (0.44) | 0.000 | < 0.001 | < 0.001 |
| **Occupation, mean (SD)** |  |  |  |  |  |  |  |
| Civil servant | 0.06 (0.23) | 0.06 (0.24) | 0.04 (0.20) | 0.05 (0.21) | <0.001 | < 0.001 | < 0.001 |
| Worker | 0.04 (0.19) | 0.04 (0.20) | 0.03 (0.16) | 0.02 (0.14) | <0.001 | < 0.001 | < 0.001 |
| Farmer | 0.39 (0.49) | 0.43 (0.50) | 0.19 (0.39) | 0.32 (0.47) | 0.000 | < 0.001 | < 0.001 |
| Freelancers | 0.02 (0.14) | 0.02 (0.15) | 0.02 (0.13) | 0.02 (0.15) | 0.002 | 0.007 | 0.661 |
| Unemployed | 0.03 (0.17) | 0.03 (0.17) | 0.05 (0.21) | 0.04 (0.19) | <0.001 | < 0.001 | 0.001 |
| Retirement | 0.12 (0.32) | 0.08 (0.27) | 0.30 (0.46) | 0.14 (0.34) | 0.000 | < 0.001 | < 0.001 |
| **Charlson score , mean (SD)** | 1.02 (1.33) | 0.92 (1.26) | 1.44 (1.55) | 1.16 (1.34) | <0.001 | 0.166 | <0.001 |
| **Surgery, mean (SD)** | 0.03 (0.18) | 0.03 (0.18) | 0.04 (0.19) | 0.03 (0.18) | 0.152 | 0.014 | 0.798 |
| **LOS, mean (SD)** | 5.85 (7.06) | 5.80 (6.98) | 5.95 (7.00) | 6.33 (8.06) | 0.272 | 0.999 | 0.810 |
| **Admission type, mean (SD)** |  |  |  |  |  |  |  |
| Emergency at arrival | 0.07 (0.25) | 0.07 (0.25) | 0.06 (0.24) | 0.05 (0.21) | <0.001 | 0.009 | < 0.001 |
| Urgent at arrival | 0.25 (0.43) | 0.23 (0.42) | 0.35 (0.48) | 0.21 (0.41) | <0.001 | < 0.001 | 0.003 |
| **Admission source, mean (SD)** |  |  |  |  |  |  |  |
| Emergency admission | 0.19 (0.39) | 0.18 (0.39) | 0.23 (0.42) | 0.16 (0.37) | <0.001 | < 0.001 | < 0.001 |
| Outpatient admission | 0.78 (0.41) | 0.78 (0.41) | 0.75 (0.43) | 0.82 (0.38) | <0.001 | < 0.001 | < 0.001 |

Abbreviations: PNA, pneumonia; FP, private for-profit; PH, public hospital; PNA, pneumonia; PNFP, private not-for-profit; SD, standard deviation; UEBMI, urban employee basic medical insurance; URBMI, urban resident basic medical insurance; LOS, length of stay; NCMS, new rural cooperative medical scheme.

^a^ *P* value was estimated by one-way ANOVA.

^b^ *P* value was estimated by paired t-test with Bonferroni adjustment.

Table A3. The characteristics for HF in Sichuan province of China during the fourth quarters of 2016-2018.

| **Variables** | All | Public | Private not-for-profit | Private for-profit | *P* for all^a^ | *P* (PNFP vs PH)^b^ | *P* (PNP vs PH)^b^ |
| --- | --- | --- | --- | --- | --- | --- | --- |
|  | (*N*=9,022) | (*N*=7,808) | (*N*=515) | (*N*=699) |  |  |  |
| **Age, mean (SD)** | 72.16 (12.04) | 72.34 (11.57) | 73.77 (13.03) | 68.93 (15.44) | <0.001 | 0.027 | < 0.001 |
| **Men, mean (SD)** | 0.47 (0.50) | 0.47 (0.50) | 0.49 (0.50) | 0.49 (0.50) | 0.459 | 0.348 | 0.377 |
| **Insurance type, mean (SD)** |  |  |  |  |  |  |  |
| UEBMI | 0.24 (0.43) | 0.22 (0.41) | 0.38 (0.49) | 0.37 (0.48) | <0.001 | < 0.001 | < 0.001 |
| URBMI | 0.34 (0.48) | 0.34 (0.47) | 0.36 (0.48) | 0.34 (0.47) | 0.671 | 0.426 | 0.743 |
| NCMS | 0.27 (0.44) | 0.29 (0.45) | 0.16 (0.37) | 0.13 (0.34) | <0.001 | < 0.001 | < 0.001 |
| **Occupation, mean (SD)** |  |  |  |  |  |  |  |
| Civil servant | 0.04 (0.19) | 0.04 (0.20) | 0.01 (0.12) | 0.01 (0.08) | <0.001 | 0.004 | < 0.001 |
| Worker | 0.01 (0.10) | 0.01 (0.10) | 0.02 (0.14) | 0.01 (0.11) | 0.080 | 0.080 | 0.616 |
| Farmer | 0.47 (0.50) | 0.50 (0.50) | 0.37 (0.48) | 0.23 (0.42) | <0.001 | < 0.001 | < 0.001 |
| Freelancers | 0.02 (0.15) | 0.01 (0.12) | 0.03 (0.17) | 0.10 (0.29) | <0.001 | 0.034 | < 0.001 |
| Unemployed | 0.03 (0.16) | 0.02 (0.15) | 0.05 (0.22) | 0.05 (0.21) | <0.001 | 0.001 | 0.003 |
| Retirement | 0.09 (0.29) | 0.08 (0.27) | 0.24 (0.43) | 0.10 (0.30) | <0.001 | < 0.001 | 0.110 |
| **Charlson score , mean (SD)** | 1.67 (1.46) | 1.59 (1.42) | 2.09 (1.59) | 2.27 (1.61) | <0.001 | < 0.001 | < 0.001 |
| **Surgery, mean (SD)** | 0.03 (0.17) | 0.03 (0.16) | 0.04 (0.19) | 0.04 (0.21) | 0.018 | 0.426 | 0.035 |
| **LOS, mean (SD)** | 10.11 (7.92) | 9.81 (6.69) | 13.48 (12.13) | 10.96 (8.22) | <0.001 | < 0.001 | < 0.001 |
| **Admission type, mean (SD)** |  |  |  |  |  |  |  |
| Emergency at arrival | 0.17 (0.37) | 0.17 (0.37) | 0.21 (0.41) | 0.12 (0.33) | <0.001 | 0.047 | 0.006 |
| Urgent at arrival | 0.29 (0.45) | 0.29 (0.45) | 0.26 (0.44) | 0.28 (0.45) | 0.262 | 0.393 | 0.455 |
| **Admission source, mean (SD)** |  |  |  |  |  |  |  |
| Emergency admission | 0.22 (0.42) | 0.22 (0.42) | 0.30 (0.46) | 0.17 (0.37) | <0.001 | < 0.001 | 0.002 |
| Outpatient admission | 0.75 (0.43) | 0.75 (0.44) | 0.69 (0.46) | 0.80 (0.40) | <0.001 | 0.023 | 0.002 |

Abbreviations: HF, heart failure; FP, private for-profit; PH, public hospital; PNA, pneumonia; PNFP, private not-for-profit; SD, standard deviation; UEBMI, urban employee basic medical insurance; URBMI, urban resident basic medical insurance; LOS, length of stay; NCMS, new rural cooperative medical scheme.

^a^ *P* value was estimated by one-way ANOVA.

^b^ *P* value was estimated by paired t-test with Bonferroni adjustment.

Table A4. The characteristics for AMI in Sichuan province of China during the fourth quarters of 2016-2018.

| Variables | All | Public | Private not-for-profit | Private for-profit | *P* for all^a^ | *P* (PNFP vs PH)^b^ | *P* (PNP vs PH)^b^ |
| --- | --- | --- | --- | --- | --- | --- | --- |
|  | (*N*=3,216) | (*N*=2,783) | (*N*=191) | (*N*=242) |  |  |  |
| **Age, mean (SD)** | 70.82 (12.12) | 70.58 (12.13) | 73.51 (11.33) | 71.53 (12.31) | 0.003 | 0.004 | 0.714 |
| **Men, mean (SD)** | 0.63 (0.48) | 0.63 (0.48) | 0.69 (0.46) | 0.60 (0.49) | 0.138 | 0.210 | 0.498 |
| **Insurance type, mean (SD)** |  |  |  |  |  |  |  |
| UEBMI | 0.23 (0.42) | 0.21 (0.41) | 0.45 (0.50) | 0.27 (0.44) | <0.001 | < 0.001 | 0.162 |
| URBMI | 0.33 (0.47) | 0.34 (0.47) | 0.20 (0.40) | 0.37 (0.48) | <0.001 | < 0.001 | 0.378 |
| NCMS | 0.28 (0.45) | 0.30 (0.46) | 0.17 (0.38) | 0.17 (0.38) | <0.001 | < 0.001 | < 0.001 |
| **Occupation, mean (SD)** |  |  |  |  |  |  |  |
| Civil servant | 0.04 (0.20) | 0.05 (0.21) | 0.03 (0.16) | 0.03 (0.17) | 0.220 | 0.583 | 0.636 |
| Worker | 0.02 (0.13) | 0.02 (0.13) | 0.03 (0.17) | 0.01 (0.09) | 0.198 | 0.530 | 0.830 |
| Farmer | 0.48 (0.50) | 0.51 (0.50) | 0.29 (0.45) | 0.30 (0.46) | <0.001 | < 0.001 | < 0.001 |
| Freelancers | 0.02 (0.13) | 0.02 (0.13) | 0.02 (0.14) | 0.01 (0.11) | 0.783 | 0.730 | 0.521 |
| Unemployed | 0.03 (0.16) | 0.03 (0.16) | 0.05 (0.21) | 0.01 (0.11) | 0.081 | 0.245 | 0.594 |
| Retirement | 0.10 (0.30) | 0.09 (0.29) | 0.30 (0.46) | 0.10 (0.30) | <0.001 | < 0.001 | 0.491 |
| **Charlson score , mean (SD)** | 1.28 (1.39) | 1.24 (1.37) | 1.73 (1.64) | 1.35 (1.33) | <0.001 | 0.999 | < 0.001 |
| **Surgery, mean (SD)** | 0.06 (0.23) | 0.05 (0.22) | 0.12 (0.33) | 0.07 (0.26) | <0.001 | < 0.001 | 0.530 |
| **LOS, mean (SD)** | 9.77 (6.08) | 9.55 (5.76) | 10.69 (7.17) | 10.02 (6.52) | <0.001 | < 0.001 | < 0.001 |
| **Admission type, mean (SD)** |  |  |  |  |  |  |  |
| Emergency at arrival | 0.38 (0.48) | 0.39 (0.49) | 0.34 (0.48) | 0.23 (0.42) | <0.001 | 0.425 | < 0.001 |
| Urgent at arrival | 0.28 (0.45) | 0.28 (0.45) | 0.34 (0.48) | 0.25 (0.43) | 0.102 | 0.258 | 0.760 |
| **Admission source, mean (SD)** |  |  |  |  |  |  |  |
| Emergency admission | 0.39 (0.49) | 0.40 (0.49) | 0.36 (0.48) | 0.24 (0.43) | <0.001 | 0.679 | < 0.001 |
| Outpatient admission | 0.58 (0.49) | 0.56 (0.50) | 0.63 (0.48) | 0.74 (0.44) | <0.001 | 0.169 | < 0.001 |

Abbreviations: AMI, acute myocardial infarction; FP, private for-profit; PH, public hospital; PNA, pneumonia; PNFP, private not-for-profit; SD, standard deviation; UEBMI, urban employee basic medical insurance; URBMI, urban resident basic medical insurance; LOS, length of stay; NCMS, new rural cooperative medical scheme.

^a^ *P* value was estimated by one-way ANOVA.

^b^ *P* value was estimated by paired t-test with Bonferroni adjustment.

Table A5. The basic characteristics of hospitals by ownership in Sichuan province of China during the fourth quarters of 2016-2018.

| Year | Ownership | Hospital grade | | | Whether general hospital | |
| --- | --- | --- | --- | --- | --- | --- |
|  |  | Grade A  (*n* (%)) | Grade B  (*n* (%)) | Non-graded  (*n* (%)) | Yes  (*n* (%)) | No  (*n* (%)) |
| 2016 | Public hospital  (N=352) | 243  (69.03) | 94  (26.70) | 15  (4.26) | 210  (59.66) | 142  (40.34) |
| 2016 | Private not-for-profit hospital  (N=88) | 19  (21.59) | 38  (43.18) | 31  (35.23) | 58  (65.91) | 30  (34.09) |
| 2016 | Private for-profit hospital  (N=92) | 18  (19.57) | 39  (42.39) | 35  (38.04) | 71  (77.17) | 21  (22.83) |
| 2017 | Public hospital  (N=354) | 249  (70.34) | 89  (25.14) | 16  (4.52) | 210  (59.32) | 144  (40.68) |
| 2017 | Private not-for-profit hospital  (N=93) | 19  (20.43) | 37  (39.78) | 37  (39.78) | 59  (63.44) | 34  (36.56) |
| 2017 | Private for-profit hospital  (N=95) | 16  (16.84) | 39  (41.05) | 40  (42.11) | 69  (72.63) | 26  (27.37) |
| 2018 | Public hospital  (N=353) | 225  (63.73) | 86  (24.36) | 42  (11.90) | 209  (59.21) | 144  (40.79) |
| 2018 | Private not-for-profit hospital  (N=90) | 17  (18.89) | 39  (43.33) | 34  (37.78) | 57  (63.33) | 33  (36.67) |
| 2018 | Private for-profit hospital  (N=95) | 15  (16.67) | 40  (42.11) | 40  (42.11) | 67  (70.53) | 28  (29.47) |

Table A6. The difference of in-hospital mortality among public, private not-for-profit, and private for-profit hospitals in Sichuan province of China during the fourth quarters of 2016-2018.

| Diseases | Public | Private not-for-profit | Private for-profit |
| --- | --- | --- | --- |
|  | (*p*-value) | (*p*-value) | (*p*-value) |
| **PNA** |  |  |  |
| Public | 1 |  |  |
| Private not-for-profit | 0.021 | 1 |  |
| Private for-profit | 0.027 | 0.956 | 1 |
| **HF** |  |  |  |
| Public | 1 |  |  |
| Private not-for-profit | 0.590 | 1 |  |
| Private for-profit | 0.403 | 0.240 | 1 |
| **AMI** |  |  |  |
| Public | 1 |  |  |
| Private not-for-profit | 0.595 | 1 |  |
| Private for-profit | 0.651 | 0.958 | 1 |

Abbreviations: AMI, acute myocardial infarction; HF, heart failure; PNA, pneumonia; coef, coefficient; CI, confidence interval; UEBMI, urban employee basic medical insurance; URBMI, urban resident basic medical insurance; NCMS, new rural cooperative medical scheme.

Adjusted for hospital type (general and non-general), hospital grade (grade A, grade B, and non-graded), volume (continuous, log-transform), gender (male and female), age (continuous, time scale), insurance type (UEBMI, NCMS, URBMI, and others), occupation (civil servant, worker, farmer, freelances, unemployed, retirement, and others), Charlson score index (continuous), surgical condition (no and yes), admission type (emergency, urgent, and elective), admission source (emergency admission, outpatient admission, and others), LOS (continuous, log-transform), year (2016,2017, and 2018), and disease subtypes (dummies).

Table A7. The relationship between the interaction of hospital ownership types and year with in-hospital mortality rate in Sichuan province of China during the fourth quarters of 2016-2018.

| **Diseases** | **Dependent variable: In-hospital mortality rate** | | |
| --- | --- | --- | --- |
|  | **PNA** | **HF** | **AMI** |
|  | **OR (95% CI)** | **OR (95% CI)** | **OR (95% CI)** |
| **Ownership** |  |  |  |
| Public | Ref. | Ref. | Ref. |
| Private not-for-profit | 1.40 (0.82, 2.38) | 0.47 (0.16, 1.43) | 1.44 (0.60, 3.46) |
| Private for-profit | 1.24 (0.56, 2.76) | 0.99 (0.30, 3.23) | 1.98 (0.70, 5.58) |
| *P* value^a^ | 0.03 | 0.52 | 0.80 |
| **Year** |  |  |  |
| 2016 | Ref. | Ref. | Ref. |
| 2017 | 1.22 (0.95, 1.56) | 1.00 (0.70, 1.42) | 0.93 (0.65, 1.33) |
| 2018 | 1.29 (0.99, 1.68) | 0.52 (0.34, 0.78)** | 0.65 (0.43, 0.99)* |
| *P* value^a^ | 0.01 | < 0.001 | 0.01 |
| **Ownership*Year** |  |  |  |
| Private not-for-profit*2017 | 1.29 (0.81, 2.04) | 2.24 (0.64, 7.83) | 0.82 (0.28, 2.41) |
| Private for-profit*2018 | 1.41 (0.86, 2.30) | 2.09 (0.53, 8.24) | 0.67 (0.22, 2.04) |
| Private not-for-profit*2017 | 1.56 (0.70, 3.46) | 1.27 (0.34, 4.76) | 0.65 (0.20, 2.04) |
| Private for-profit*2018 | 1.31 (0.59, 2.93) | 1.46 (0.39, 5.40) | 0.40 (0.12, 1.36) |
| *P* value^a^ | 0.54 | 0.75 | 0.63 |
| **Control variables** |  |  |  |
| Hospital characteristics | Yes | Yes | Yes |
| Patient characteristics | Yes | No | Yes |

Abbreviations: AMI, acute myocardial infarction; HF, heart failure; PNA, pneumonia; CI, confidence interval; OR, odds ratio.

All models were adjusted for hospital type (general and non-general), hospital grade (grade A, grade B, and non-graded), volume (continuous, log-transform), gender (male and female), age (continuous, time scale), insurance type (UEBMI, NCMS, URBMI, and others), occupation (civil servant, worker, farmer, freelances, unemployed, retirement, and others), Charlson score index (continuous), surgical condition (no and yes), admission type (emergency, urgent, and elective), admission source (emergency admission, outpatient admission, and others), LOS (continuous, log-transform), and disease subtypes (dummies).

^a^ *P* for value was used to estimate the overall effects of ownership, year and interaction of ownership and year on in-hospital mortality using Wald tests.

Table A8. The difference of medical expenses among the public, private not-for-profit, and private for-profit hospitals in Sichuan province of China during the fourth quarters of 2016-2018.

| Diseases | Public | Private not-for-profit | Private for-profit |
| --- | --- | --- | --- |
|  | (*p*-value) | (*p*-value) | (*p*-value) |
| **PNA** |  |  |  |
| Public | 1 |  |  |
| Private not-for-profit | <0.001 | 1 |  |
| Private for-profit | <0.001 | 0.764 | 1 |
| **HF** |  |  |  |
| Public | 1 |  |  |
| Private not-for-profit | 0.036 | 1 |  |
| Private for-profit | 0.281 | 0.270 | 1 |
| **AMI** |  |  |  |
| Public | 1 |  |  |
| Private not-for-profit | 0.392 | 1 |  |
| Private for-profit | 0.865 | 0.543 | 1 |

Abbreviations: AMI, acute myocardial infarction; HF, heart failure; PNA, pneumonia; coef, coefficient; CI, confidence interval; UEBMI, urban employee basic medical insurance; URBMI, urban resident basic medical insurance; NCMS, new rural cooperative medical scheme.

Adjusted for hospital type (general and non-general), hospital grade (grade A, grade B, and non-graded), volume (continuous, log-transform), gender (male and female), age (continuous, time scale), insurance type (UEBMI, NCMS, URBMI, and others), occupation (civil servant, worker, farmer, freelances, unemployed, retirement, and others), Charlson score index (continuous), surgical condition (no and yes), admission type (emergency, urgent, and elective), admission source (emergency admission, outpatient admission, and others), death (no and yes), LOS (continuous, log-transform), year (2016,2017, and 2018), and disease subtypes (dummies).

Table A9. The relationship between the interaction of hospital ownership types and year with medical expenses in Sichuan province of China during the fourth quarters of 2016-2018.

| **Diseases** | **Dependent variable: medical expenses** | |  |
| --- | --- | --- | --- |
|  | **PNA** | **HF** | **AMI** |
|  | **Coef (95% CI)** | **Coef (95% CI)** | **Coef (95% CI)** |
| **Ownership** |  |  |  |
| Public | Ref. | Ref. | Ref. |
| Private not-for-profit | 0.07 (0.03, 0.11)** | 0.04 (-0.06, 0.14) | 0.04 (-0.13, 0.22) |
| Private for-profit | 0.05 (0.01, 0.10)* | -0.02 (-0.13, 0.09) | 0.06 (-0.13, 0.25) |
| *P* value^a^ | < 0.001 | 0.11 | 0.69 |
| **Year** |  |  |  |
| 2016 | Ref. | Ref. | Ref. |
| 2017 | -0.02 (-0.03, -0.01)*** | -0.01 (-0.03, 0.02) | 0.04 (-0.01, 0.09) |
| 2018 | 0.02 (0.01, 0.03)*** | 0.01 (-0.01, 0.04) | 0.05 (-0.01, 0.11) |
| *P* value^a^ | < 0.001 | 0.11 | 0.11 |
| **Ownership*Year** |  |  |  |
| Private not-for-profit*2017 | 0.07 (0.05, 0.09)*** | 0.06 (-0.04, 0.16) | -0.11 (-0.32, 0.09) |
| Private not-for-profit*2018 | 0.06 (0.04, 0.08)*** | 0.08 (-0.03, 0.18) | 0.12 (-0.08, 0.32) |
| Private for-profit*2017 | 0.05 (0.02, 0.08)** | 0.08 (-0.03, 0.18) | -0.02 (-0.22, 0.17) |
| Private for-profit*2018 | 0.07 (0.04, 0.10)*** | 0.06 (-0.05, 0.16) | -0.07 (-0.27, 0.13) |
| *P* value^a^ | < 0.001 | 0.35 | 0.13 |
| **Control variables** |  |  |  |
| Hospital characteristics | Yes | Yes | Yes |
| Patient characteristics | Yes | No | Yes |

Abbreviations: AMI, acute myocardial infarction; HF, heart failure; PNA, pneumonia; CI, confidence interval; coef, coefficient; OR, odds ratio.

All models were adjusted for hospital type (general and non-general), hospital grade (grade A, grade B, and non-graded), volume (continuous, log-transform), gender (male and female), age (continuous, time scale), insurance type (UEBMI, NCMS, URBMI, and others), occupation (civil servant, worker, farmer, freelances, unemployed, retirement, and others), Charlson score index (continuous), surgical condition (no and yes), admission type (emergency, urgent, and elective), admission source (emergency admission, outpatient admission, and others), death (no and yes), LOS (continuous, log-transform), and disease subtypes (dummies).

^a^ *P* for value was used to estimate the overall effects of ownership, year and interaction of ownership and year on medical expenses using wald tests.

Table A10. The subgroup analyses for the relationship betwee hospital ownership types and in-hospital mortality in Sichuan province of China during the fourth quarters of 2016-2018.

| Characteristics | **PNA** | |  | **HF** | |  | **AMI** | |  |
| --- | --- | --- | --- | --- | --- | --- | --- | --- | --- |
|  | Private not-for-profit | Private for-profit | *P* for interaction | Private not-for-profit | Private for-profit | *P* for interaction | Private not-for-profit | Private for-profit | *P* for interaction |
|  | OR (95% CI) | OR (95% CI) |  | OR (95% CI) | OR (95% CI) |  | OR (95% CI) | OR (95% CI) |  |
| **Age** |  |  | 0.15 |  |  | 0.53 |  |  | 0.38 |
| Age <= 60 | 0.61 (0.20, 1.89) | 1.25 (0.40, 3.88) |  | 0.57 (0.12, 2.80) | 0.21 (0.04, 1.06) |  | 0.12 (0.01, 2.49) | 1.05 (0.18, 5.94) |  |
| Age > 60 | 1.93 (1.21, 3.09) | 1.77 (1.10, 2.86) |  | 0.87 (0.45, 1.67) | 1.44 (0.78, 2.66) |  | 1.36 (0.76, 2.43) | 1.20 (0.65, 2.20) |  |
| **Gender** |  |  | 0.35 |  |  | 0.43 |  |  | 0.13 |
| Male | 1.56 (0.98, 2.51) | 1.77 (1.09, 2.89) |  | 0.70 (0.32, 1.53) | 0.86 (0.39, 1.90) |  | 1.16 (0.55, 2.41) | 1.86 (0.88, 3.95) |  |
| Female | 2.02 (1.06, 3.87) | 1.51 (0.75, 3.04) |  | 1.19 (0.51, 2.78) | 1.97 (0.92, 4.21) |  | 1.55 (0.63, 3.80) | 0.51 (0.19, 1.33) |  |
| **Insurance type** |  |  | 0.21 |  |  | 0.36 |  |  | 0.02 |
| UEBMI | 2.06 (1.15, 3.71) | 1.26 (0.67, 2.38) |  | 0.94 (0.41, 2.16) | 1.52 (0.65, 3.51) |  | 0.49 (0.21, 1.13) | 0.87 (0.32, 2.38) |  |
| URBMI | 1.53 (0.72, 3.26) | 2.75 (1.29, 5.85) |  | 1.05 (0.39, 2.81) | 2.63 (1.34, 5.18) |  | 1.69 (0.56, 5.11) | 0.94 (0.37, 2.43) |  |
| NCMS | 0.55 (0.11, 2.62) | 1.30 (0.40, 4.29) |  | 1.21 (0.12, 11.96) | 0.27 (0.03, 2.73) |  | 3.71 (1.08, 12.73) | 2.37 (0.62, 9.10) |  |
| Others | 0.92 (0.45, 1.89) | 2.96 (1.36, 6.45) |  | 0.46 (0.09, 2.21) | 1.10 (0.27, 4.40) |  | 0.51 (0.09, 2.77) | 1.37 (0.30, 6.29) |  |
| **Year** |  |  | 0.52 |  |  | 0.71 |  |  | 0.62 |
| 2016 | 1.64 (0.77, 3.47) | 1.82 (0.61, 5.43) |  | 0.52 (0.12, 2.23) | 1.15 (0.20, 6.81) |  | 1.23 (0.40, 3.78) | 2.21 (0.58, 8.43) |  |
| 2017 | 1.61 (0.80, 3.27) | 3.47 (1.61, 7.45) |  | 1.74 (0.65, 4.65) | 1.15 (0.20, 6.82) |  | 1.01 (0.44, 2.36) | 1.08 (0.47, 2.52) |  |
| 2018 | 1.54 (0.85, 2.79) | 1.53 (0.84, 2.78) |  | 0.90 (0.30, 2.68) | 1.15 (0.20, 6.83) |  | 1.36 (0.44, 4.21) | 0.80 (0.26, 2.45) |  |

Abbreviations: AMI, acute myocardial infarction; HF, heart failure; PNA, pneumonia; OR, odds ratio; CI, confidence interval; UEBMI, urban employee basic medical insurance; URBMI, urban resident basic medical insurance; NCMS, new rural cooperative medical scheme.

Adjusted for hospital type (general and non-general), hospital grade (grade A, grade B, and non-graded), volume (continuous, log-transform), gender (male and female), age (continuous, time scale), insurance type (UEBMI, NCMS, URBMI, and others), occupation (civil servant, worker, farmer, freelances, unemployed, retirement, and others), Charlson score index (continuous), surgical condition (no and yes), admission type (emergency, urgent, and elective), admission source (emergency admission, outpatient admission, and others), LOS (continuous, log-transform), year (2016,2017, and 2018), and disease subtypes (dummies).

- indicating no analyzed results because the sample size was too small to get valid results.

* indicating 0.01≤ p-value <0.05

** indicating 0.001≤ p-value <0.01

*** indicating p-value <0.001

Table A11. The subgroup analyses for the relationship between the hospital ownership types and medical expense in Sichuan province of China during the fourth quarters of 2016-2018.

| Characteristics | **PNA** | |  | **HF** | |  | **AMI** | |  |
| --- | --- | --- | --- | --- | --- | --- | --- | --- | --- |
|  | Private not-for-profit | Private for-profit | *P* for interaction | Private not-for-profit | Private for-profit | *P* for interaction | Private not-for-profit | Private for-profit | *P* for interaction |
|  | Coef (95% CI) | Coef (95% CI) |  | Coef (95% CI) | Coef (95% CI) |  | Coef (95% CI) | Coef (95% CI) |  |
| **Age** |  |  | < 0.001 |  |  | 0.90 |  |  | 0.20 |
| Age <= 60 | 0.08 (0.02, 0.13) | 0.12 (0.07, 0.17) |  | 0.04 (-0.12, 0.20) | -0.01 (-0.15, 0.13) |  | -0.06 (-0.34, 0.22) | -0.03 (-0.28, 0.22) |  |
| Age > 60 | 0.13 (0.08, 0.18) | 0.12 (0.07, 0.16) |  | 0.08 (0.00, 0.16) | 0.05 (-0.03, 0.13) |  | 0.08 (-0.05, 0.20) | 0.05 (-0.07, 0.18) |  |
| **Gender** |  |  | 0.64 |  |  | 0.42 |  |  | 0.20 |
| Male | 0.10 (0.05, 0.16) | 0.10 (0.05, 0.15) |  | 0.11 (0.01, 0.21) | 0.08 (-0.02, 0.17) |  | 0.06 (-0.07, 0.20) | -0.04 (-0.18, 0.10) |  |
| Female | 0.11 (0.07, 0.16) | 0.14 (0.09, 0.18) |  | 0.04 (-0.05, 0.14) | 0.02 (-0.07, 0.11) |  | 0.09 (-0.10, 0.27) | 0.16 (-0.01, 0.33) |  |
| **Insurance type** |  |  | < 0.001 |  |  | 0.34 |  |  | 0.23 |
| UEBMI | 0.13 (0.07, 0.18) | 0.09 (0.04, 0.13) |  | 0.10 (-0.02, 0.22) | 0.00 (-0.12, 0.11) |  | 0.03 (-0.14, 0.20) | 0.09 (-0.10, 0.28) |  |
| URBMI | 0.10 (0.03, 0.18) | 0.21 (0.14, 0.29) |  | 0.06 (-0.06, 0.19) | 0.16 (0.02, 0.29) |  | 0.13 (-0.09, 0.34) | 0.04 (-0.14, 0.22) |  |
| NCMS | 0.11 (0.03, 0.19) | 0.09 (0.03, 0.15) |  | -0.01 (-0.18, 0.16) | 0.02 (-0.14, 0.17) |  | 0.03 (-0.25, 0.31) | 0.12 (-0.12, 0.36) |  |
| Others | 0.06 (-0.02, 0.14) | 0.14 (0.06, 0.21) |  | 0.15 (-0.03, 0.33) | 0.03 (-0.13, 0.18) |  | -0.05 (-0.33, 0.22) | -0.22 (-0.47, 0.02) |  |
| **Year** |  |  | < 0.001 |  |  | 0.32 |  |  | 0.13 |
| 2016 | 0.11 (0.03, 0.19) | 0.16 (0.07, 0.25) |  | 0.07 (-0.08, 0.21) | 0.05 (-0.11, 0.22) |  | 0.15 (-0.06, 0.37) | 0.07 (-0.18, 0.31) |  |
| 2017 | 0.10 (0.02, 0.18) | 0.14 (0.06, 0.23) |  | 0.08 (-0.06, 0.22) | 0.07 (-0.07, 0.21) |  | -0.08 (-0.25, 0.10) | 0.06 (-0.11, 0.23) |  |
| 2018 | 0.06 (-0.02, 0.14) | 0.17 (0.09, 0.25) |  | 0.02 (-0.09, 0.12) | 0.10 (-0.01, 0.20) |  | 0.14 (-0.04, 0.33) | 0.04 (-0.13, 0.21) |  |

Abbreviations: AMI, acute myocardial infarction; HF, heart failure; PNA, pneumonia; coef, coefficient; CI, confidence interval; UEBMI, urban employee basic medical insurance; URBMI, urban resident basic medical insurance; NCMS, new rural cooperative medical scheme.

Adjusted for hospital type (general and non-general), hospital grade (grade A, grade B and non-graded), volume (continuous, log-transform), gender (male and female), age (continuous, time scale), insurance type (UEBMI, NCMS, URBMI, and others), occupation (civil servant, worker, farmer, freelances, unemployed, retirement, and others), Charlson score index (continuous), surgical condition (no and yes), admission type (emergency, urgent, and elective), admission source (emergency admission, outpatient admission, and others), death (no and yes), LOS (continuous, log-transform), year (2016, 2017, and 2018), and disease subtypes (dummies).

* indicating 0.01 ≤ *p*-value < 0.05

** indicating 0.001 ≤ *p*-value < 0.01

*** indicating *p*-value < 0.001

Table A12. The relationship between whether private or not or whether for-profit or not and in-hospital mortality in Sichuan province of China during the fourth quarters of 2016-2018.

| Diseases | Before matching | After matching |
| --- | --- | --- |
|  | OR (95% CI) | OR (95% CI) |
| **PNA** |  |  |
| **Sample size** | 51,933 | 47,192 |
| **Hospital ownership** |  |  |
| Public | 1 | 1 |
| Private | 1.68 (1.08, 2.62) * | 1.97 (1.18, 3.27) *** |
| **Whether for-profit** |  |  |
| No | 1 | 1 |
| Yes | 0.99 (0.63, 1.56) | 1.01 (0.59, 1.73) |
| **HF** |  |  |
| **Sample size** | 9,022 | 5,630 |
| **Hospital ownership** |  |  |
| Public | 1 | 1 |
| Private | 0.86 (0.47, 1.57) | 1.21 (0.52, 2.79) |
| **Whether for-profit** |  |  |
| No | 1 | 1 |
| Yes | 1.51 (0.77, 2.97) | 1.36 (0.55, 3.39) |
| **AMI** |  |  |
| **Sample size** | 3,216 | 1,561 |
| **Hospital ownership** |  |  |
| Public | 1 | 1 |
| Private | 1.20 (0.67, 2.15) | 0.93 (0.44, 1.97) |
| **Whether for-profit** |  |  |
| No | 1 | 1 |
| Yes | 0.99 (0.49, 1.97) | 1.02 (0.45, 2.31) |
| **Control variables** | Yes | Yes |

Abbreviations: AMI, acute myocardial infarction; HF, heart failure; PNA, pneumonia; OR, odds ratio; CI, confidence interval; UEBMI, urban employee basic medical insurance; URBMI, urban resident basic medical insurance; NCMS, new rural cooperative medical scheme.

Adjusted for hospital type (general hospital and non-general hospital), hospital grade (grade A, grade B, and non-graded), volume (continuous, log-transform), gender (male and female), age (continuous, time scale), insurance type (UEBMI, NCMS, URBMI and others), occupation (civil servant, worker, farmer, freelances, unemployed, retirement, and others), Charlson score index (continuous), surgical condition (no and yes), admission type (emergency, urgent, and elective), admission source (emergency admission, outpatient admission, and others), LOS (continuous, log-transform), year (2016,2017, and 2018) and disease subtypes (dummies).

* indicating 0.01 ≤ *p*-value < 0.05

** indicating 0.001 ≤ *p*-value < 0.01

*** indicating *p*-value < 0.001

Table A13. The relationship between whether private or not or whether for-profit or not and medical expenses in Sichuan province of China during the fourth quarters of 2016-2018.

| **Diseases** | **Before matching** | **After matching** |
| --- | --- | --- |
|  | **Coef (95% CI)** | **Coef (95% CI)** |
| **PNA** |  |  |
| **Sample size** | 51,933 | 47,192 |
| **Hospital ownership** |  |  |
| Public | 1 | 1 |
| Private | 0.11 (0.06, 0.15) *** | 0.10 (0.06, 0.14) *** |
| **Whether for-profit** |  |  |
| No | 1 | 1 |
| Yes | 0.01 (-0.03, 0.04) | -0.01 (-0.04, 0.02) |
| **HF** |  |  |
| **Sample size** | 9,022 | 5,630 |
| **Hospital ownership** |  |  |
| Public | 1 | 1 |
| Private | 0.09 (0.01, 0.17) * | 0.02 (-0.06, 0.11) |
| **Whether for-profit** |  |  |
| No | 1 | 1 |
| Yes | -0.05 (-0.13, 0.04) | -0.07 (-0.15, 0.02) |
| **AMI** |  |  |
| **Sample size** | 3,126 | 1,561 |
| **Hospital ownership** |  |  |
| Public | 1 | 1 |
| Private | 0.05 (-0.07, 0.18) | 0.03 (-0.12, 0.18) |
| **Whether for-profit** |  |  |
| No | 1 | 1 |
| Yes | -0.04 (-0.18, 0.09) | -0.04 (-0.21, 0.12) |
| **Control variables** | Yes | Yes |

Abbreviations: AMI, acute myocardial infarction; HF, heart failure; PNA, pneumonia; coef, coefficient; CI, confidence interval; UEBMI, urban employee basic medical insurance; URBMI, urban resident basic medical insurance; NCMS, new rural cooperative medical scheme.

Adjusted for hospital type (general hospital and non-general hospital), hospital grade (grade A, grade B, and non-graded), volume (continuous, log-transform), gender (male and female), age (continuous, time scale), insurance type (UEBMI, NRCMS, URBMI and others), occupation (civil servant, worker, farmer, freelances, unemployed, retirement, and others), Charlson score index (continuous), surgical condition (no and yes), admission type (emergency, urgent, and elective), admission source (emergency admission, outpatient admission and others), death ( no and yes), LOS (continuous, log-transform), year (2016,2017, and 2018), and disease subtypes (dummies).

* indicating 0.01≤ *p*-value <0.05

** indicating 0.001≤ *p*-value <0.01

*** indicating *p*-value <0.001

Table A14. Associations between hospital ownership types and LOS in Sichuan province of China during the fourth quarters of 2016-2018.

| **Diseases** | **Dependent variable: log (LOS)** | | |
| --- | --- | --- | --- |
|  | Model 1 | Model 2 | Model 3 |
|  | OR (95% CI) | OR (95% CI) | OR (95% CI) |
| **PNA** |  |  |  |
| Public | Ref. | Ref. | Ref. |
| Private not-for-profit | 0.03 (-0.02, 0.08) | 0.03 (-0.02, 0.08) | 0.02 (-0.03, 0.07) |
| Private for-profit | 0.02 (-0.03, 0.06) | 0.02 (-0.03, 0.06) | -0.01 (-0.06, 0.03) |
| **Random parts** |  |  |  |
| Between-state variance | 0.21 | 0.21 | 0.19 |
| Intra Class Correlation (ICC) | 0.27 | 0.27 | 0.26 |
| N_Hospitals_ | 503 | 503 | 503 |
| N_Individuals_ | 51,933 | 51,933 | 51,933 |
| **HF** |  |  |  |
| Public | Ref. | Ref. | Ref. |
| Private not-for-profit | 0.18 (0.07, 0.28)** | 0.21 (0.09, 0.32)*** | 0.15 (0.04, 0.26)** |
| Private for-profit | 0.08 (-0.01, 0.18) | 0.11 (-0.01, 0.21) | 0.06 (-0.04, 0.17) |
| **Random parts** |  |  |  |
| Between-state variance | 0.27 | 0.25 | 0.24 |
| Intra Class Correlation (ICC) | 0.27 | 0.26 | 0.25 |
| N_Hospitals_ | 419 | 419 | 419 |
| N_Individuals_ | 9,022 | 9,022 | 9,022 |
| **AMI** |  |  |  |
| Public | Ref. | Ref. | Ref. |
| Private not-for-profit | 0.04 (-0.17, 0.26) | 0.10 (-0.13, 0.33) | -0.01 (-0.22, 0.22) |
| Private for-profit | 0.16 (-9.05, 0.36) | 0.18 (-0.05, 0.40) | 0.13 (-0.09, 0.35) |
| **Random parts** |  |  |  |
| Between-state variance | 0.38 | 0.37 | 0.37 |
| Intra Class Correlation (ICC) | 0.26 | 0.26 | 0.27 |
| N_Hospitals_ | 352 | 352 | 352 |
| N_Individuals_ | 3,216 | 3,216 | 3,216 |
| **Control variables** |  |  |  |
| Hospital characteristics^1^ | No | Yes | Yes |
| Patient characteristics^2^ | No | No | Yes |
| Year dummies | Yes | Yes | Yes |

Abbreviations: AMI, acute myocardial infarction; HF, heart failure; PNA, pneumonia; OR, odds ratio; CI, confidence interval; UEBMI, urban employee basic medical insurance; URBMI, urban resident basic medical insurance; NCMS, new rural cooperative medical scheme. Ref: Reference category.

^1^ Hospital characteristics included hospital type (general and non-general), hospital grade (grade A, grade B and non-graded), and volume (continuous, log-transform).

^2^ Patient characteristics included gender (male and female), age (continuous, time scale), insurance type (UEBMI, NCMS, URBMI, and others), occupation (civil servant, worker, farmer, freelances, unemployed, retirement, and others), Charlson score index (continuous), surgical condition (no and yes), admission type (emergency, urgent, and elective), admission source (emergency admission, outpatient admission, and others), and disease subtypes (dummies).

* indicating 0.01 ≤ *p*-value < 0.05

** indicating 0.001 ≤ *p*-value < 0.01

*** indicating *p*-value < 0.001
